# Wistar Rats Resistant to the Hypertensive Effects of Ouabain Exhibit Enhanced Cardiac Vagal Activity and Elevated Plasma Levels of Calcitonin Gene-Related Peptide

**DOI:** 10.1371/journal.pone.0108909

**Published:** 2014-10-03

**Authors:** Elham Ghadhanfar, Maie Al-Bader, Marian Turcani

**Affiliations:** Department of Physiology, Faculty of Medicine, Kuwait University, Kuwait City, Kuwait; Univeristy of Glasgow, United Kingdom

## Abstract

Ouabain is a cardiac glycoside produced in the adrenal glands and hypothalamus. It affects the function of all cells by binding to Na^+^/K^+^-ATPase. Several lines of evidence suggest that endogenous ouabain could be involved in the pathogenesis of essential (particularly, salt-sensitive) hypertension. However, information regarding the postulated hypertensive effect of the long-term administration of low-dose exogenous ouabain is inconsistent. This study was designed to help settle this controversy through the use of telemetric monitoring of arterial blood pressure and to elucidate the ouabain-induced alterations that could either promote or prevent hypertension. Ouabain (63 and 324 µg/kg/day) was administered subcutaneously to male Wistar rats. Radiotelemetry was used to monitor blood pressure, heart rate and measures of cardiovascular variability and baroreflex sensitivity. The continuous administration of ouabain for 3 months did not elevate arterial blood pressure. The low-frequency power of systolic pressure variability, urinary excretion of catecholamines, and cardiovascular response to restraint stress and a high-salt diet as well as the responsiveness to α1-adrenergic stimulation were all unaltered by ouabain administration, suggesting that the activity of the sympathetic nervous system was not increased. However, surrogate indices of cardiac vagal nerve activity based on heart rate variability were elevated. Molecular remodeling in mesenteric arteries that could support the development of hypertension (increased expression of the genes for the Na^+^/Ca^2+^ exchanger and Na^+^/K^+^-ATPase α2 isoform) was not evident. Instead, the plasma level of vasodilatory calcitonin gene-related peptide (CGRP) significantly rose from 55 (11, SD) in the control group to 89 (20, SD) pg/ml in the ouabain-treated rats (P_Tukey's_ = 18.10^−5^). These data show that long-term administration of exogenous ouabain does not necessarily cause hypertension in rodents. The augmented parasympathetic activity and elevated plasma level of CGRP could be linked to the missing hypertensive effect of ouabain administration.

## Introduction

Ouabain is a cardiotonic glycoside secreted by the adrenal glands and hypothalamus. The binding of ouabain to its receptor, Na^+^/K^+^-ATPase, inhibits ATPase activity and initiates several cellular signaling pathways [1]. Ouabain could play important roles in the maintenance of sodium and body fluid homeostasis and in the regulation of arterial pressure; consequently, ouabain may also affect the pathogenesis of hypertension [2].

The permanent elevation of arterial pressure during the chronic administration of low-dose exogenous ouabain (Table S1) strongly supports the notion that endogenous ouabain plays a major pathophysiological role in essential hypertension. If ouabain is indeed a causative agent in essential hypertension, its application to normotensive animals in pathophysiologically relevant amounts should unequivocally elevate arterial pressure.

However, an increase in arterial pressure in response to the chronic application of exogenous ouabain has not always been reported. At least nine independent studies of appropriate duration and adequate ouabain dosing have not detected any arterial blood pressure elevation (Table S2). Even authors that have repeatedly documented ouabain-induced hypertension have noted in some reports that the arterial blood pressure response to exogenous ouabain was variable, i.e., ouabain was causing hypertension in some rats but not in others (Table S3). Nevertheless, these negative reports have mostly been ignored, and the prevailing opinion is that endogenous ouabain is an important factor in the pathogenesis of hypertension [2]–[5].

Despite rather compelling evidence at the cellular and molecular levels that supports the postulated causal relationship between elevated ouabain and elevated arterial blood pressure, the fact that exogenous ouabain causes hypertension has not yet been proven. For instance, arterial pressure elevation associated with chronic low-dose ouabain has not been confirmed with telemetric monitoring, the recommended gold-standard method for blood pressure measurement in animals [6]. Thus, it is possible that the reported discrepancies in arterial blood pressure response to chronic low-dose ouabain could be related to limitations associated with the tail-cuff measurements of blood pressure. Moreover, the absence of a hypotensive effect of the ouabain antagonist rostafuroxin in an OASIS-HT trial does not support a major pathophysiological role for endogenous ouabain in essential hypertension [7].

The main goal of this study was to use radiotelemetric blood pressure monitoring to test whether the chronic administration of low-dose exogenous ouabain causes hypertension. Furthermore, we examined ouabain-induced changes in autonomic nervous system activity, the stress response, and the expression of genes that have been postulated to be critically involved in ouabain-dependent hypertension. Because ouabain plasma levels are particularly elevated in patients with salt-sensitive hypertension [8], we explored whether the application of ouabain is associated with increased sensitivity of blood pressure to salt. Although we carefully designed our experimental protocol to mimic published research showing a hypertensive effect of exogenous ouabain, we were unable to confirm this finding. We hypothesized that increased secretion of some vasodilators could prevent the hypertensive effect of ouabain and found that the plasma level of calcitonin gene-related peptide (CGRP) was elevated in ouabain-treated rats.

## Materials and Methods

For a detailed description of the experimental procedures, please see the supporting information (Methods S1).

### Ethics statement

The animal care and animal-associated procedures were performed in accordance with the National Institutes of Health Guide for the Care and Use of Laboratory Animals (8^th^ edition), which has been adopted as the standard for animal care and use by the Animal Resource Center at Kuwait University. The study was approved by the Animal Welfare Committee at Kuwait University Health Sciences Center. The animals were released from Kuwait University's Animal Resource Center only after all requirements were fulfilled.

### Experimental animals and the administration of ouabain

Inbred 12-week-old male Wistar rats (Animal Resource Center, Kuwait University) were implanted with telemetric transmitters and were randomly (http://www.graphpad.com/quickcalcs/randomize1.cfm) divided into control and ouabain treatment groups (10 rats/group). In the control group, one animal died from unknown causes 11 days after the implantation. After recovery from the implantation surgery (minimum 3 weeks), baseline telemetric monitoring was performed, and placebo or ouabain release pellets (Innovative Research of America, Sarasota, FL) were implanted subcutaneously on the lateral side of the neck. Rats aged 21–23 weeks weighing 391 (21) g received two doses of ouabain, 63 (3) and 324 (21) µg of ouabain/kg/day (the numbers in parentheses represent the standard deviations). The actual average dose per body weight was calculated by taking into account changes in body weight during the experiment.

Exact information about ouabain doses is not available in many published articles, e.g., only the amount of ouabain administered to an animal is given, but the weight of the animal is not reported. Thus, it was difficult to make a clear conclusion regarding the postulated dose-dependency of ouabain's hypertensive effects [9] and to choose the most appropriate dosing. We decided to administer 60 µg ouabain/kg/day, the dose most commonly used when ouabain-induced hypertension was documented, and 320 µg ouabain/kg/day, the highest dose repeatedly associated with blood pressure elevation in response to ouabain (Tables S1–S3).

### Telemetric monitoring and data analysis

Telemetrically transmitted data were collected with a data acquisition system (Dataquest ART 3.1, Data Sciences, St. Paul, MN). Parameters, such as systolic, diastolic, pulse and mean aortic blood pressures as well as heart rate, pre-ejection time, respiratory rate, and body temperature, were extracted from the original waveforms (arterial pressure, ECG, temperature, and locomotor activity) as the mean values of 10-s intervals with Dataquest ART Analysis 4.3 software (Data Sciences, St. Paul, MN). The circadian rhythmic pattern in the arterial blood pressure oscillations was estimated with cosinor analysis (Chronos-Fit software, P. Zuther and B. Lemmer, http://www.ma.uni-heidelberg.de/inst/phar/forschungLemmer.html, 2004). Linear and non-linear measures characterizing the short-term heart rate and blood pressure variability as well as the baroreflex sensitivity were calculated with WinCPRS software (Absolute Aliens Oy, Turku, Finland).

### Assessment of the activity of the sympathetic nervous system

Blood pressure variability, pre-ejection time, ganglionic blockade, α1-adrenergic stimulation, restraint, low/high-salt diet, and urinary catecholamine excretion were used to assess the activity of the sympathetic nervous system.

### Reverse transcription quantitative polymerase chain reaction

RNA was extracted with TRIzol Reagent (Invitrogen, NY), and the RNA's integrity was checked using 1% agarose gel electrophoresis in MOPS buffer with ethidium bromide staining. After treatment with DNase, the samples were reverse transcribed. qPCR with TaqMan hydrolysis probes was conducted using an Applied Biosciences thermal cycler model 7500. Expression of the following genes was assessed: the α1, α2, and α3 isoforms of Na^+^/K^+^-ATPase; Na^+^/Ca^2+^ exchanger 1; nitric oxide synthase 1–3; and cyclooxygenase 1–2.

### Immunoassays

Plasma and urine samples were analyzed using commercially available kits by following the manufacturer's procedures: CGRP (A05482, SPI Bio, Bertin Pharma, Montigny le Bretonneux, France), cyclic guanosine monophosphate (cGMP; ST-505 Cell Biolabs, San Diego, CA), epinephrine and norepinephrine (BA E-5400, LDN Labor Diagnostika Nord, Nordhorn, Germany), and nitrate/nitrite (780001, Cayman, Ann Arbor, MI).

### Liquid chromatography-tandem mass spectrometry (LC/MS)

The ouabain content and potential digoxin contamination in the implantable pellets and rat chow was checked with LC/MS using Quattro LC equipment (Micromass, Manchester, UK).

### Statistical analysis

The data are presented as the means with the corresponding standard deviations or 95% confidence intervals in brackets. Values of P<0.05 were considered significant. A multivariate approach to repeated measures was generally used. A description of specific statistical methods is given in the figure and table legends and in the supplemental methods section.

## Results

### Ouabain did not increase arterial blood pressure

**Figure 1:**
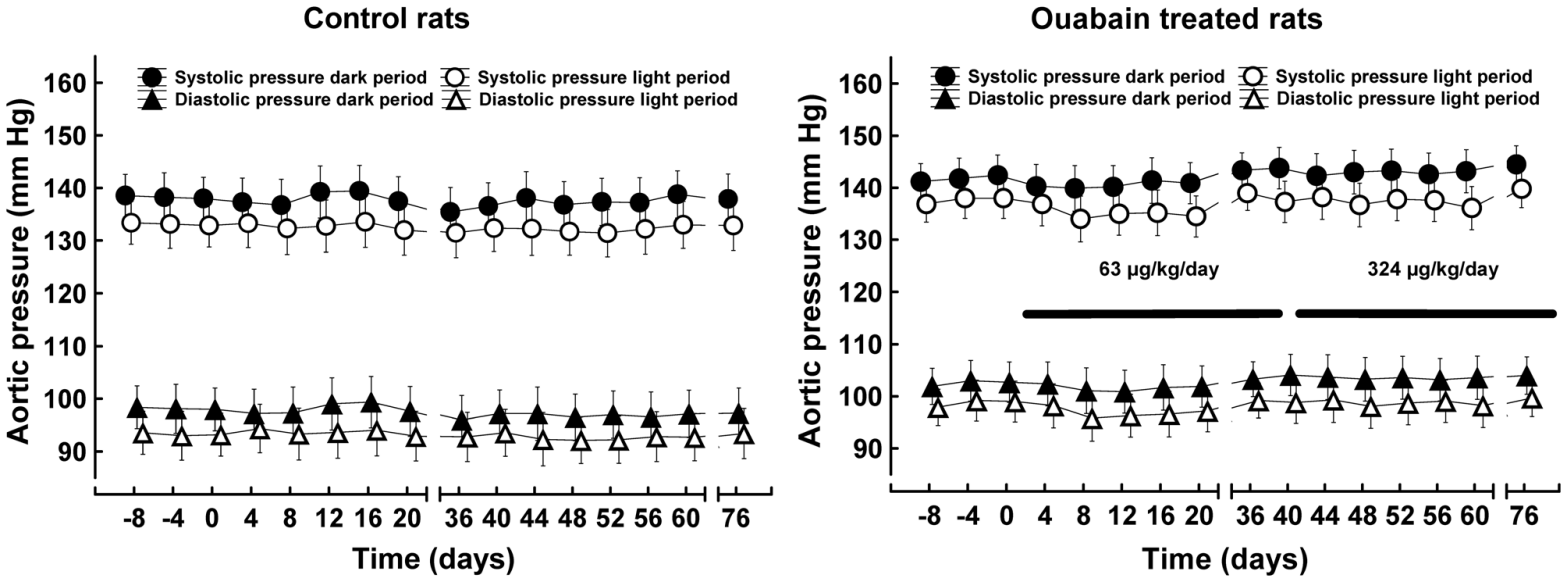
Effect of ouabain on arterial blood pressure. The data points represent the 12-hour means; the vertical bars denote the 95% CIs (confidence intervals). Pharmacological, salt sensitivity, stress and tail-cuff simulation tests were performed during days 20–40 and 60–80. Control rats, n = 9; ouabain-treated rats, n = 10. Data were analyzed with MANOVA for repeated measures and the multivariate Wilk's test (between-within design; 2 levels of the main effect “group” x 2 levels of the main effect “illumination” x 16 levels of the main effect “time/ouabain treatment”). F, multivariate F-test value, subscripts are degrees of freedom; P, probability. Systolic pressure: main effect “group”, i.e., control vs. ouabain group, F_1,17_ = 1.95, P = 0.18; main effect “time” F_15,3_ = 1.51, P = 0.415; main effect “illumination” F_1,17_ = 143, P<10^−6^; no significant interactions between the main effects were present. Diastolic pressure: main effect “group” F_1,17_ = 4.39, P = 0.052; main effect “time” F_15,3_ = 5.91, P = 0.08; main effect “illumination” F_1,17_ = 229, P<10^−6^; no significant interactions between the main effects were present.

Arterial blood pressure was telemetrically monitored for 24 hours at 4-day intervals, but no hypertensive or hypotensive effect of ouabain was observed at any dose (Figure 1). To exclude the possibility that the average arterial pressure values did not increase significantly because only some rats were sensitive to the hypertensive effect of ouabain, we checked the pressure changes for each individual rat in which an ouabain pellet had been implanted. None of the rats exhibited an arterial pressure elevation of more than 3 mm Hg over the 11-week period of treatment (Figure S1).

We also did not find any significant difference in the circadian rhythms of the mean arterial pressure between the control and ouabain-treated rats. Within-group analysis identified only a small, significant quadratic trend toward lower-pressure values (F_1,17_ = 6.3, P<0.02) during the light period in the ouabain-treated rats (Table S4). Several other parameters (heart rate, pre-ejection time, respiratory rate and body temperature) that could be affected by ouabain-induced alterations in autonomic nervous system activity were assessed telemetrically, but no ouabain effect could be identified (Figure S2).

### Ouabain elevated surrogate measures of cardiac vagal nerve activity

**Figure 2:**
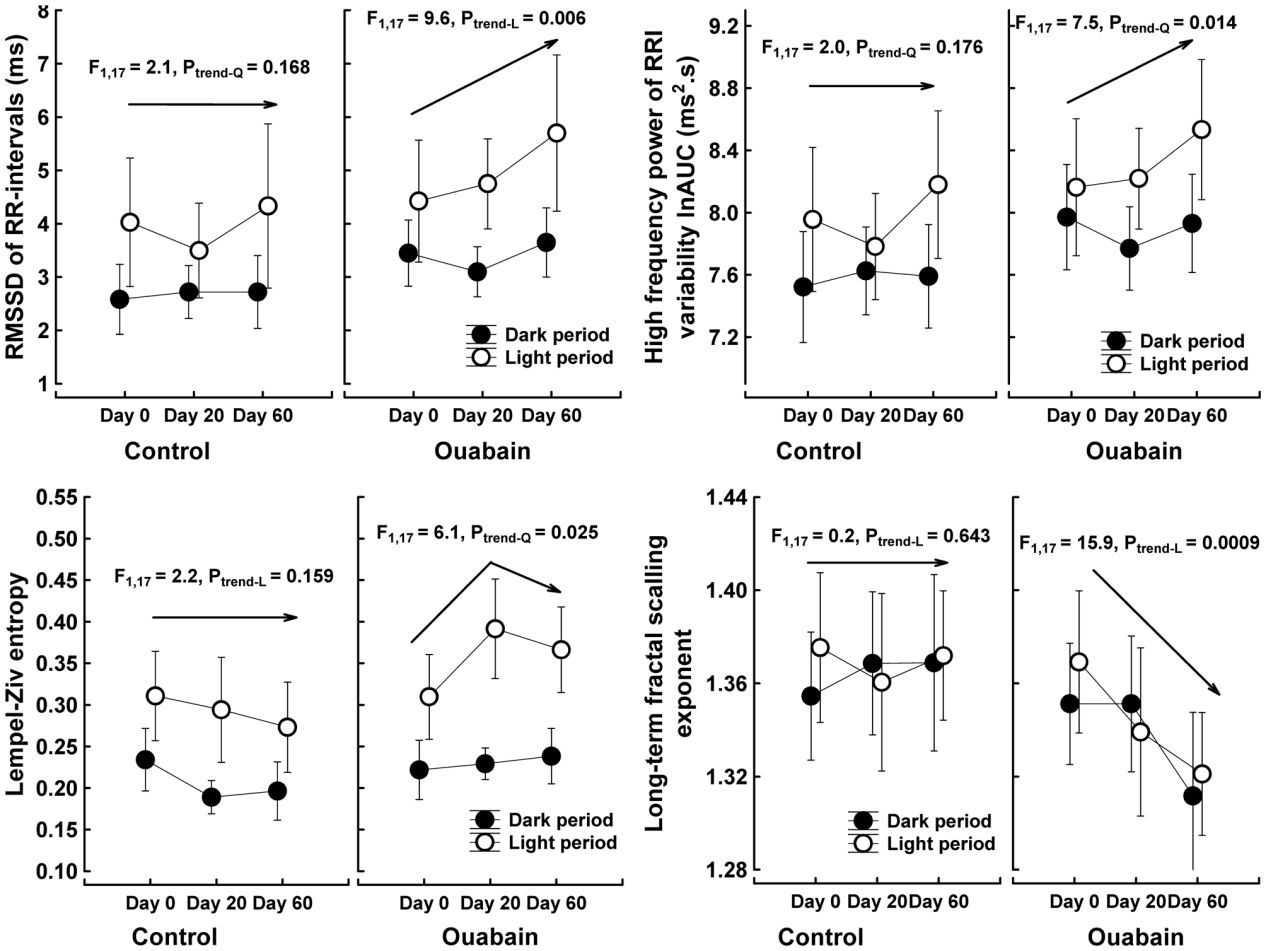
Surrogate measures of cardiac vagal nerve activity derived from the RR-interval variability. The symbols represent the means, and the vertical bars indicate the 95% CIs; n = 9 control rats, n = 10 ouabain-treated rats. Day 0, rats were not treated with ouabain; Day 20, rats were treated for 20 days with 63 µg ouabain/kg/day; Day 60, rats were treated for 40 days with 63 µg ouabain/kg/day and then 20 days with 324 µg ouabain/kg/day. RMSSD, square root of the mean sum of the squares of differences between adjacent RR-intervals; AUC, area under curve; ln, natural logarithm; F, univariate F-test value, subscripts are degrees of freedom; P_trend_, probability that a relationship (L-linear, Q-quadratic) between the time and RRI variability is a coincidence. The arrows denote the trend analysis for the light period. The main effects and their interactions were tested with repeated measures MANOVA and the multivariate Wilk's test; the between groups main effect “group” was tested with the univariate ANOVA (between-within design; 2 levels of main effect “group” x 2 levels of main effect “illumination” x 3 levels of main effect “time/ouabain treatment”; the MANOVA results are in Tables S3 and S5. Significant simple effects were identified with the contrast and trend analyses of *a priori* defined comparisons.

As expected, most of the heart rate variability parameters exhibited significant differences between the dark and light periods (Tables S5–S6). Planned comparisons with contrast and trend analysis showed that ouabain influenced the vagal regulation of the sino-atrial node during the light (inactive) period. The dose/time-dependent increases in the root mean square of the successive differences (RMSSD) and the high-frequency power of RR-interval variability suggest that ouabain stimulated cardiac vagal activity during the light period. Elevations in Lempel-Ziv entropy could be interpreted as reduced predictability in the heart rate oscillations, which is consistent with a more intensive cardiac vagal drive. This possibility is further supported by the reduced correlations between the RR intervals that occurred over long time scales (Figure 2).

Several different mathematical approaches were used to assess the sensitivity of the spontaneous baroreflex. All baroreflex sensitivity measures showed strong dependence on dark-light cycles, with higher values during the light period. However, no significant effect of ouabain was detected (Table S7). Nevertheless, rejection of the null-hypothesis (i.e., ouabain treatment has no effect on baroreflex sensitivity) was only borderline (P = 0.058) for the alpha index calculated in the high-frequency range. This measure should particularly reflect the vagal component of the baroreflex [10].

### No signs of sympathetic overactivity during the administration of ouabain

Ouabain did not augment the low-frequency power of systolic pressure variability, an index of vascular sympathetic drive [11]. Although some blood pressure variability parameters were modified by illumination differently in the control and ouabain-treated groups (i.e., there was a significant illumination x group interaction) and although the illumination effect changed over time (i.e., there was a significant time x illumination interaction), the identified significant simple contrasts were not related to the ouabain treatment (Tables S8–S9).

**Figure 3:**
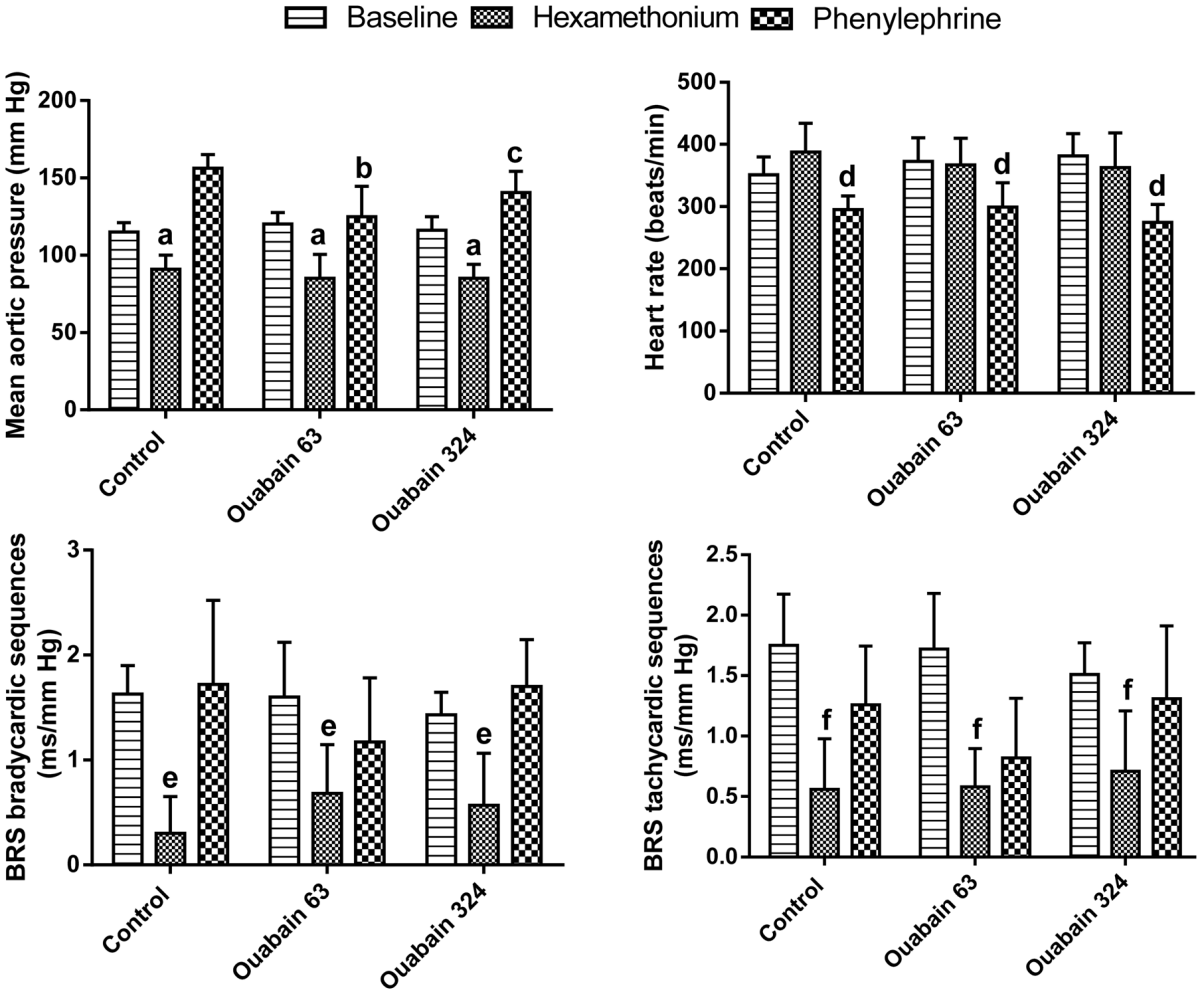
Hemodynamic effects of ganglionic blockade and α1-adrenergic stimulation. The columns represent the mean values of 3 minute-long intervals before the injection of hexamethonium bromide (30 mg/kg s.c.), i.e., baseline and during the maximum blood pressure response (i.e., between ca. the 12^th^–15^th^ min after the application of hexamethonium and between ca. the 27^th^–30^th^ min after the administration of phenylephrine hydrochloride (3 mg/kg s.c.)); the vertical bars denote the 95% confidence intervals; n = 10 rats. Control, pharmacological test performed before the implantation of the ouabain pellets; Ouabain 63, test performed when rats were treated for 21 days with 63 µg ouabain/kg/day; Ouabain 324, test applied to rats treated for 40 days with 63 µg ouabain/kg/day and then for 21 days with 324 µg ouabain/kg/day. The data were analyzed with repeated measures MANOVA and the multivariate Wilk's test in a 3 (ouabain treatment) x 3 (pharmacological intervention) design followed by the contrast and trend analysis; a, significant contrast analysis “baseline” versus “hexamethonium,” F_1,9_ = 40, P = 14.10^−5^; b, significant contrast analysis “control“ versus “ouabain 63” after phenylephrine, F_1,9_ = 21.7, P = 0.0012; c, significant contrast analysis “control“ versus “ouabain 324” after phenylephrine, F_1,9_ = 5.1, P = 0.0508; d, significant contrast analysis “hexamethonium” versus “phenylephrine”, F_1,9_ = 29, P = 44.10^−5^; e, significant contrast analysis “baseline” versus “hexamethonium”, F_1,9_ = 16.1, P = 27.10^−5^. Baroreflex sensitivity - tachycardic sequences: interaction F_4,6_ = 1.3, P = 0.359; main effect “ouabain treatment”, F_2,8_ = 0.44, P = 0.658; main effect “pharmacological intervention”, F_2,8_ = 24.1, P = 41.10^−5^; f, significant contrast analysis “baseline” versus “hexamethonium”, F_1,9_ = 53.6, P = 45.10^−6^.

In addition to assessment of the low-frequency power of systolic pressure variability, ganglionic blockade with hexamethonium was used to visualize the contribution of the autonomic nervous system to arterial blood pressure regulation. Subsequent administration of phenylephrine (an α-1 adrenergic agonist) was used to estimate the function of the postsynaptic cardiovascular α-1 adrenergic mechanism. Hexamethonium significantly reduced the arterial blood pressure to a similar extent before and during ouabain treatment (Figure 3). Consecutive injection of phenylephrine induced hypertensive and bradycardic responses, which were associated with a patchy return of the baroreflex sensitivity to baseline values (Figure 3). Treatment with a lower ouabain dose diminished the arterial blood pressure elevation after the stimulation of α-1 adrenergic receptors (F_1,9_ = 21.7; P = 12.10^−4^). After treatment with a higher ouabain dose, the reduction in the phenylephrine-elicited hypertensive response was just above the significance limit (F_1,9_ = 5.1; P = 0.0508). Moreover, trend analysis showed a significant declining quadratic trend in the arterial blood pressure elevation in response to the α-1 adrenergic stimulation during ouabain administration (F_1.9_ = 9.7; P = 0.012).

The cardiovascular response to a 30-minute-long restraint in plexiglass cylinders and the subsequent recovery period also remained unchanged during ouabain treatment (Figure S3, Tables S10–S11).

### Rats with implanted ouabain-release pellets did not exhibit increased arterial-pressure salt sensitivity

**Figure 4:**
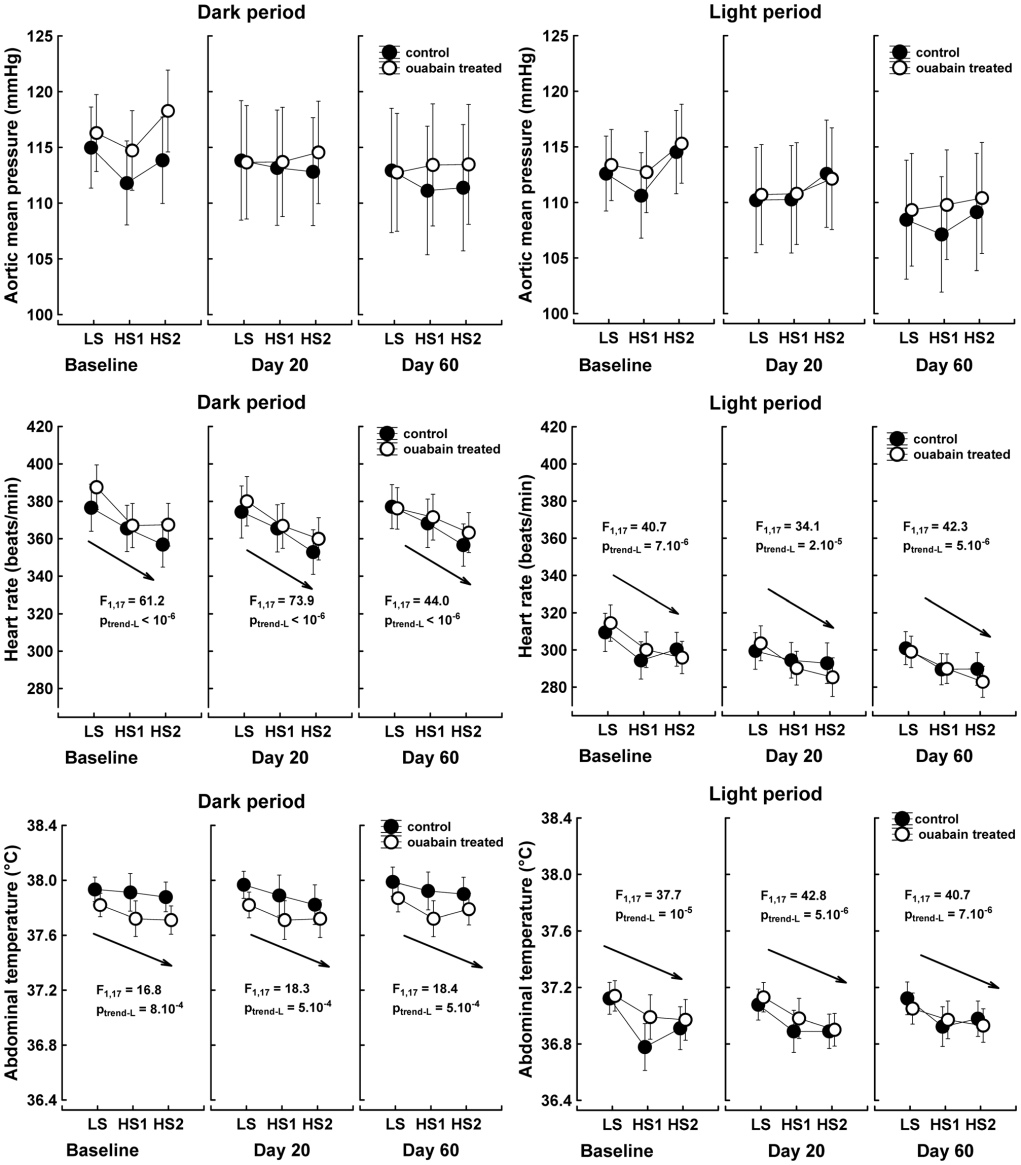
Mean aortic blood pressure, heart rate, and abdominal temperature response to increased NaCl intake. The data points represent the 12-hour means of values measured during the dark and light periods; the vertical bars denote the 95% confidence intervals, n = 9 control rats; n = 10 ouabain-treated rats. LS, low salt intake; HS1, 1^st^ day of high salt intake; HS2, 2^nd^ day of high salt intake. Baseline, salt test performed before the implantation of pellets; Day 20, rats were treated for 27–29 days with 63 µg ouabain/kg/day; Day 60, rats were treated for 40 days with 63 µg ouabain/kg/day and then for 27–29 days with 324 µg ouabain/kg/day. The data were analyzed with repeated measures MANOVA and the multivariate Wilk's test in a 2 (group) x 3 (time/ouabain treatment) x 3 (salt intake) x 2 (illumination) design. The arrows denote the linear trend analysis that was common for both groups. F, univariate F-test value, subscripts are degrees of freedom. P_trend-L_, probability that a linear relationship between time and the analyzed parameters is due to chance.

A short-term elevation of the chow NaCl content from 0.08% to 8% was associated with declines in heart rate (main effect “salt intake”: F_2,16_ = 40.9, P<10^−6^) and abdominal temperature (main effect “salt intake”: F_2,16_ = 57.2, P<10^−6^) during the dark and light periods in both experimental groups. However, the arterial pressure did not change in the control or ouabain-treated rats (Figure 4).

**Figure 5:**
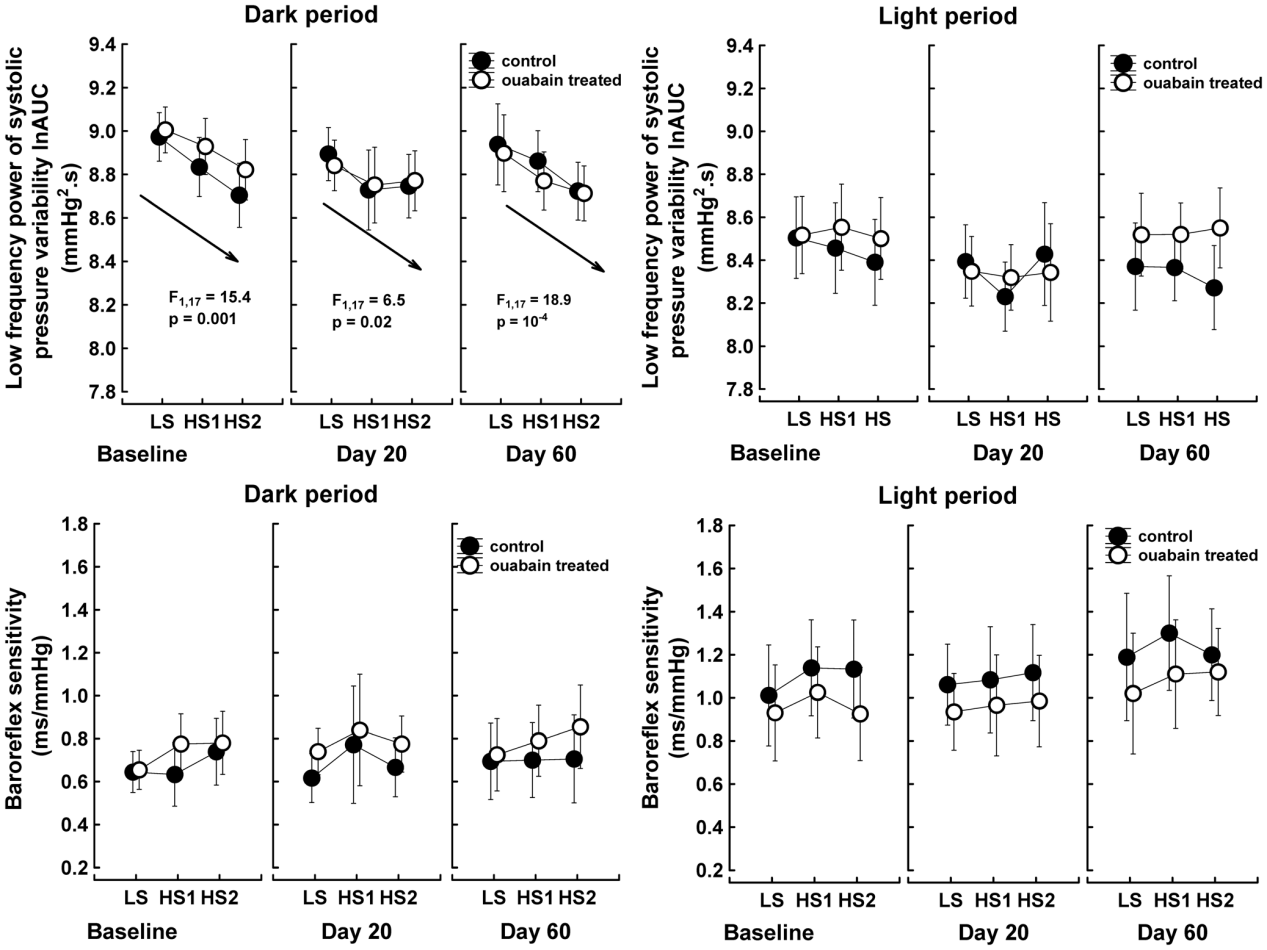
Low-frequency power of the systolic pressure variability and baroreflex sensitivity response to increased NaCl intake. The baroreflex sensitivity was estimated as the square root of the ratio of RR-interval and systolic pressure powers calculated over those frequency ranges where the coherence was >0.5 and the phase angle <0. The data points represent the means of two 35-min intervals selected in the middle of the dark and light periods; the vertical bars denote 95% confidence intervals, n = 9 control rats; n = 10 ouabain-treated rats. LS, low salt intake; HS1, 1^st^ day of high salt intake; HS2, 2^nd^ day of high salt intake. Baseline, salt test performed before the implantation of the ouabain pellets; Day 20, rats were treated for 27–29 days with 63 µg ouabain/kg/day; Day 60, rats were treated for 40 days with 63 µg ouabain/kg/day and then for 27–29 days with 324 µg ouabain/kg/day. Data were analyzed with repeated measures MANOVA and the multivariate Wilk's test in a 2 (group) x 3 (time/ouabain treatment) x 3 (salt intake) x 2 (illumination) between-within design. Arrows denote the linear trend analysis common for both groups. F, univariate F-test value, subscripts are the degrees of freedom; P_trend_, probability that a linear relationship between time and analyzed parameters is due to chance.

**Figure 6:**
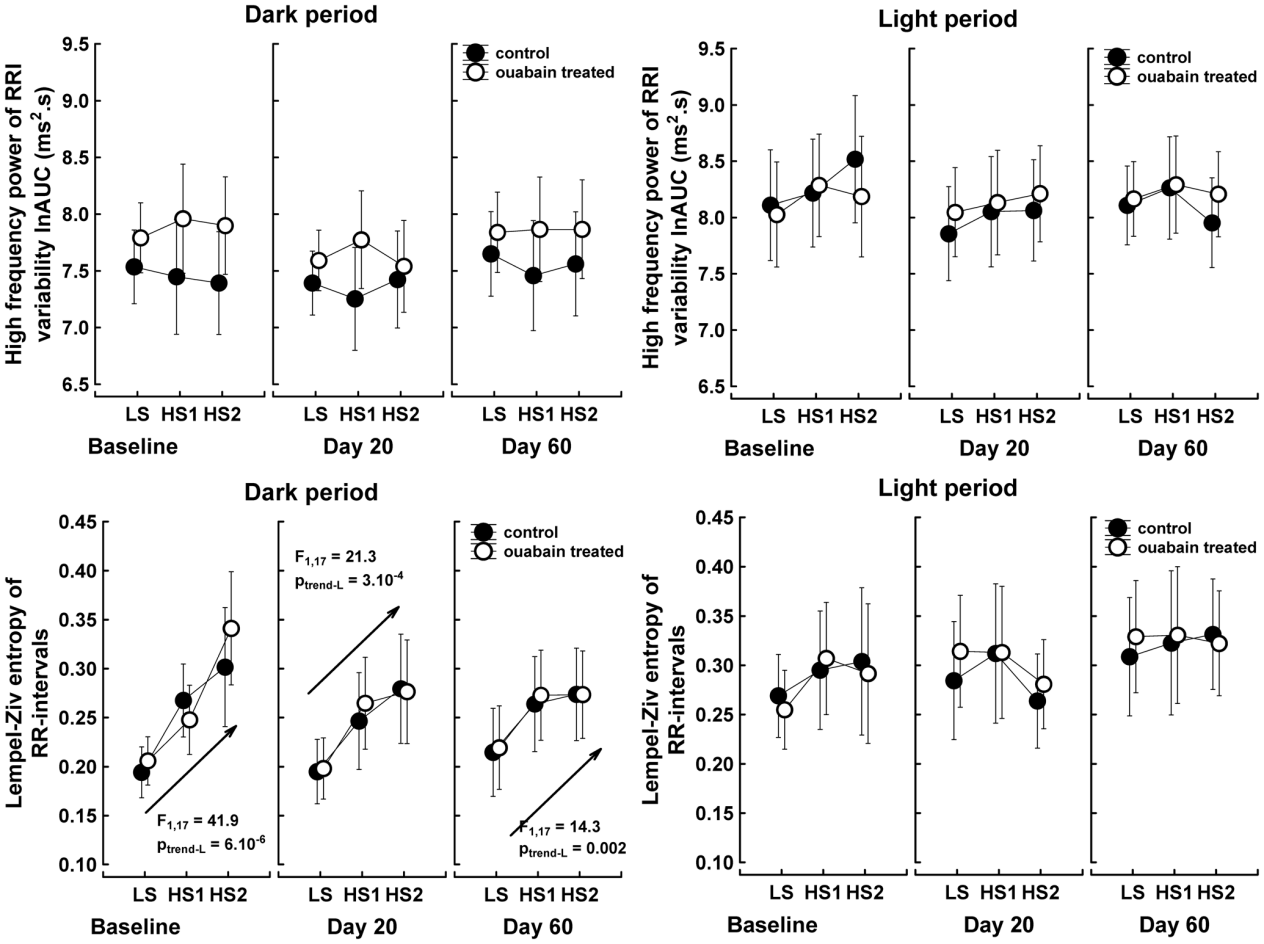
High-frequency power and Lempel-Ziv entropy of the RR-interval variability changes in response to increased NaCl intake. The data points represent the means of three 35-min intervals selected in the middle of the dark and light periods; the vertical bars denote 95% confidence intervals, n = 9 control rats; n = 10 ouabain-treated rats. LS, low salt intake; HS1, 1^st^ day of high salt intake; HS2, 2^nd^ day of high salt intake. Baseline, salt test performed before the implantation of the ouabain pellets; Day 20, rats were treated for 27–29 days with 63 µg ouabain/kg/day, Day 60, rats were treated for 40 days with 63 µg ouabain/kg/day and then for 27–29 days with 324 µg ouabain/kg/day. Data were analyzed with repeated measures MANOVA and the multivariate Wilk's test in a 2 (group) x 3 (time/ouabain treatment) x 3 (salt intake) x 2 (illumination) between-within design. The arrows denote the linear trend analysis common for both groups. F, univariate F-test value, subscripts are the degrees of freedom; P_trend_, probability that a linear relationship between time and analyzed parameters is due to chance.

As expected, the high-salt intake significantly decreased the sympathetic drive to arterioles, as reflected by the reduced low-frequency power of systolic pressure variability (main effect “salt intake”: F_2,16_ = 8.9, P = 0.003). This sympatholytic effect of the high-salt intake was present only during the dark period and was not altered by ouabain treatment (Figure 5). In contrast, the expected salt-dependent increase in baroreflex sensitivity was not observed (Figure 5). The parasympathetically driven high-frequency power of RR-interval variability also remained steady. However, the nonlinear surrogate measure of cardiac vagal activity, the Lempel-Ziv entropy of RR-intervals, gradually increased in response to the high-salt intake during the dark period in both the control and ouabain-treated rats (main effect “salt intake”: F_2,16_ = 29.4, P = 4.10^−6^). Thus, the elevated unpredictability of RR-interval fluctuations suggests an increased vagal contribution to heart rate regulation during the dark period when the animals were on a high-salt diet (Figure 6).

**Figure 7:**
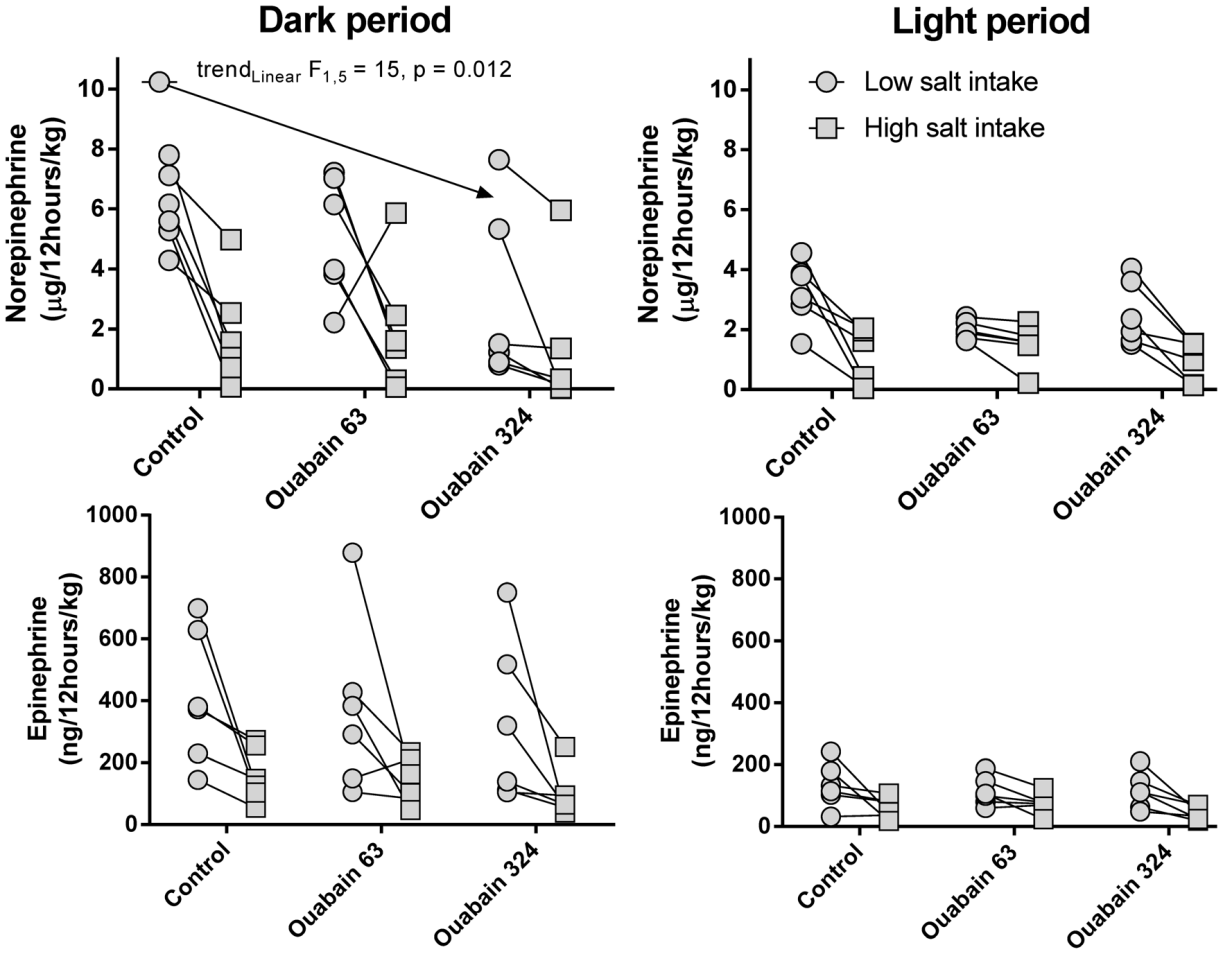
Urine excretion of catecholamines during the periods of low and high salt intake. Control, salt sensitivity test performed before the implantation of the ouabain pellets; Ouabain 63, salt sensitivity test applied to rats treated for 27–29 days with 63 µg ouabain/kg/day; Ouabain 324, test applied to rats treated for 40 days with 63 µg ouabain/kg/day and then for 27–29 days with 324 µg ouabain/kg/day. Data were analyzed with repeated measures MANOVA and the multivariate Wilk's test in a 3 (ouabain treatment) x 2 (salt intake) x 2 (illumination) design; n = 6 rats. The planned trend and contrast analysis was applied if the Wilk's test was significant (P<0.05). Norepinephrine: main effect “ouabain treatment,” F_2,4_ = 7.78, P = 0.042; main effect “salt intake,” F_1,5_ = 27.8, P = 0.003; main effect “illumination,” F_1,5_ = 7.29, P = 0.043; no interactions were significant. The arrow indicates a significant linear declining trend for norepinephrine urine excretion during the low salt intake of the dark period. Epinephrine: main effect “ouabain treatment,” F_2,4_ = 1.31, P = 0.36; main effect “salt intake,” F_1,5_ = 6.89, P = 0.047; main effect “illumination,” F_1,5_ = 16.1, P = 0.01; no interactions were significant.

The rats showed increased excretion of catecholamines when on a low-salt diet, particularly during the dark period. However, the capacity to elevate catecholamine excretion was highly variable (Figure 7). Despite this variability, ouabain dose dependently reduced norepinephrine excretion during the dark period and the low-salt diet (linear trend F_1,5_ = 15, P = 0.012).

### Ouabain did not amplify Na^+^/Ca^2+^ exchanger gene expression in the mesenteric arteries

In the mesenteric (resistance) arteries of the ouabain-treated animals, the expression of the α2 isoform of the Na^+^/K^+^-ATPase gene was depressed, and the expression of the Na^+^/Ca^2+^ exchanger gene remained unchanged. The expressions of the α1 and α3 isoforms of the Na^+^/K^+^-ATPase gene were not altered (Table 1). In the aorta (conductance vessel), ouabain significantly reduced the expression of all 3 isoforms of Na^+^/K^+^-ATPase. However, the expression of the Na^+^/Ca^2+^ exchanger gene was upregulated (Table 1). No significant changes in cyclooxygenase 1 or 2 gene expression were detected in the mesenteric arteries or in the aorta (Table 1).

**Table 1:** Effect of ouabain treatment (324 µg/kg/day) on gene expression in the mesenteric arteries and the aorta.

Gene	Mesenteric arteries			Aorta		
	Fold change	95% CI	P	Fold change	95% CI	P
**Na^+^/Ca^2+^ exchanger-1**	1.42	−2.67 to 3.28	0.277	2.67	1.50 to 5.71	<10^−3^
**α1 Na^+^/K^+^-ATPase**	1.29	−5.13 to 4.89	0.594	−2.38	−3.36 to −1.72	0.006
**α2 Na^+^/K^+^-ATPase**	−1.58	−2.51 to 1.57	0.046	−2.92	−4.24 to −1.98	0.008
**α3 Na^+^/K^+^-ATPase**	2.99	−5.56 to 35.11	0.207	−3.12	−6.71 to −1.70	0.029
**COX1**	−1.52	−71.43 to 40.07	0.710	1.24	−3.39 to 5.60	0.761
**COX2**	−1.08	−1.22 to 1.04	0.058	1.62	−1.45 to 3.93	0.305
**NOS1**	1.07	−4.41 to 3.42	0.874	−2.67	−4.10 to −1.61	0.014
**NOS3**	1.03	−1.72 to 1.42	0.858	−2.94	−6.29 to 1.71	<10^−3^
**NOS2**	not detectable					

COX1, cyclooxygenase 1 (constitutive); COX2; cyclooxygenase 2 (inducible); NOS1, nitric oxide synthase 1 (neuronal); NOS2, nitric oxide synthase 2 (inducible); NOS3, nitric oxide synthase 3 (endothelial); CI, confidence interval; P, probability. The null hypothesis was tested with the pair-wise fixed reallocation randomization test, and the 95% CI was estimated with the bootstrapping technique; n = 5.

### Ouabain elevated the plasma level of CGRP

**Figure 8:**
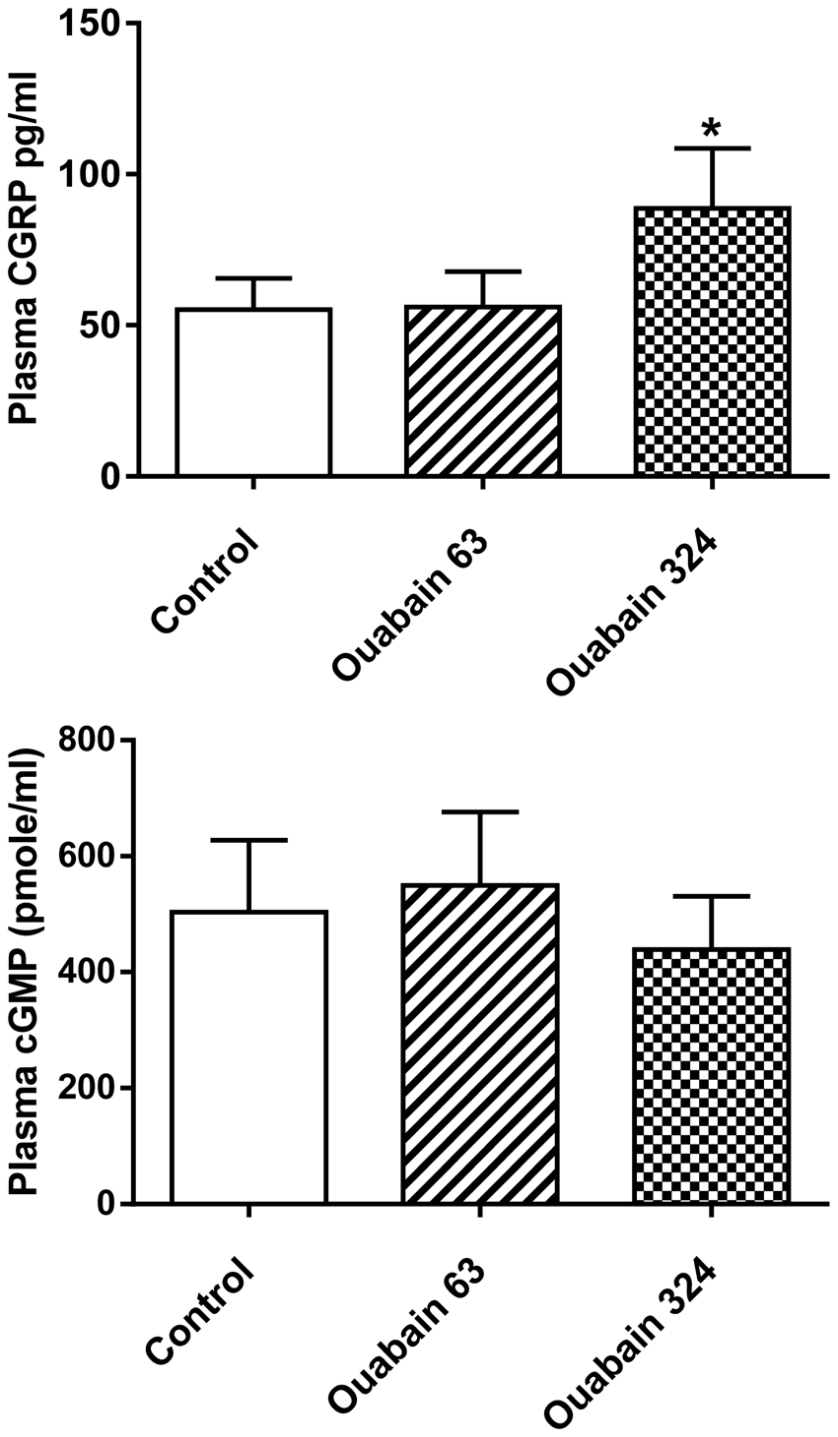
Effect of ouabain on the plasma levels of CGRP and cGMP. The data are presented as the means (SDs); n = 10. Control, rats with implanted placebo pellets; Ouabain 63, rats treated for 40 days with 63 µg ouabain/kg/day, Ouabain 324, rats treated for 40 days with 63 µg ouabain/kg/day and then for 40 days with 324 µg ouabain/kg/day. The statistical analysis was performed with one-way ANOVA and Tukey's post hoc multiple comparison test; CGRP: F_2,27_ = 17, P = 17.10^−6^; *P_Tukey's_ = 18.10^−5^ vs. control; cGMP: F_2,27_ = 1.192, P = 0.319).

An ouabain dose of 324 µg/kg/day significantly elevated the plasma level of CGRP, but no increase was found in the rats treated with 63 µg ouabain/kg/day (Figure 8). Figure 8 also shows that ouabain administration did not cause changes in the plasma level of cGMP, a second messenger of several vasodilators.

**Figure 9:**
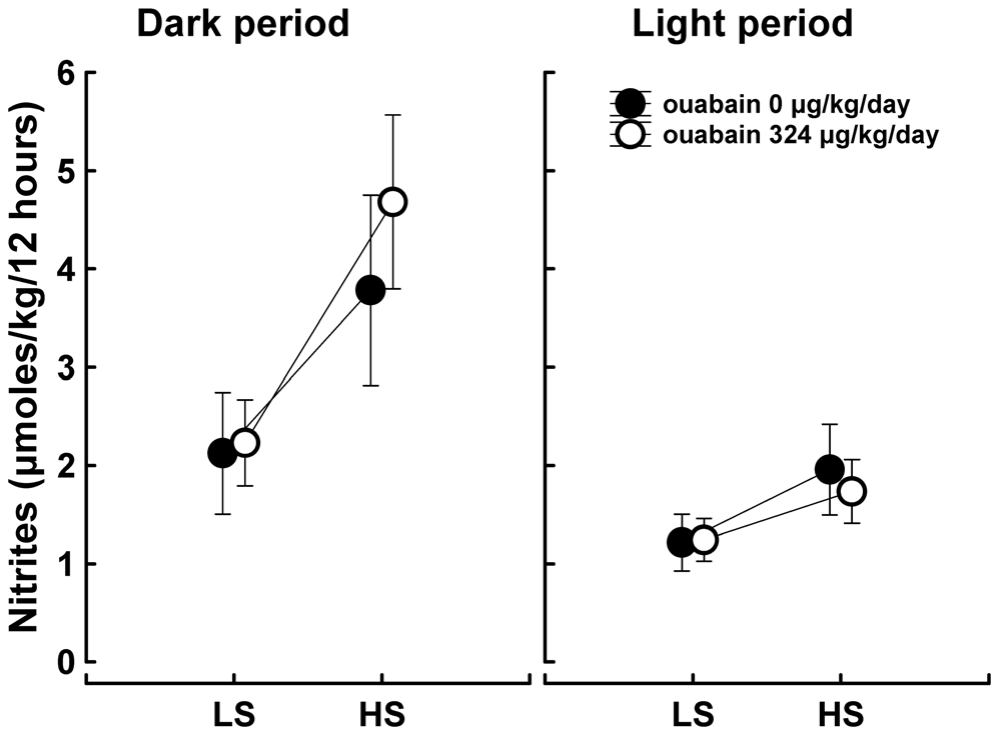
Nitrite/nitrate urine excretion. The symbols represent the means; the vertical bars indicate the 95% CIs; n = 9; LS, low salt intake; HS, high salt intake. Nitrite/nitrate urine excretion was measured before ouabain pellet implantation (ouabain 0 µg/kg/day) and after the treatment for 40 days with 63 µg ouabain/kg/day and then for 27–29 days with 324 µg ouabain/kg/day (ouabain 324 µg/kg/day). The main effects and their interactions were tested with repeated measures ANOVA (within-subjects design with 2 levels of the main effect “ouabain treatment” x 2 levels of the main effect “salt intake” x 2 levels of the main effect “illumination.” Main effect “ouabain treatment”: F_1,8_ = 1.29, P = 0.288; main effect “salt intake”: F_1,8_ = 93.61, P = 10^−5^; main effect “illumination”: F_1,8_ = 50, P = 26.10^−5^. Interaction “ouabain treatment x salt intake”: F_1,8_ = 1.42, P = 0.267; interaction “ouabain treatment x illumination”: F_1,8_ = 10.81, P = 0.011; interaction “salt intake x illumination”: F_1,8_ = 22.16, P = 15.10^−4^; interaction “ouabain treatment x salt intake x illumination”: F_1,8_ = 3.67, P = 0.092.

The urinary excretion rate of nitrites/nitrates was measured as an indirect index of nitric oxide synthesis. The urinary excretion of nitrites/nitrates was higher during the dark period than during the light period. The excretion was also stimulated by the high-salt intake, but the treatment with ouabain had no effect (Figure 9). The expression of nitric oxide synthase genes was unchanged in the mesenteric arteries but was downregulated in the aorta (Table 1).

## Discussion

The discovery of endogenous cardiotonic steroids revived interest in a half-century-old hypothesis that postulated an important role for an endogenous ouabain-like substance, i.e., a putative natriuretic and vasoconstrictory hormone, in the pathogenesis of essential hypertension [12]. A key element of this hypothesis is that low doses of exogenous ouabain, when administered chronically, elevate arterial pressure.

### Arterial blood pressure elevation during the long-term administration of low-dose ouabain could not be corroborated

Hypertensive effects of exogenous ouabain have been observed in some studies (Table S1) but not in others (Table S2). Some studies have reported variable results (Table S3). Our telemetrically recorded arterial pressure data demonstrated that the chronic administration of ouabain did not lead to hypertension in male Wistar rats (Figure 1). This finding supports the existence of resistance to the putative hypertensive effect of ouabain described in some reports (Tables S2–S3). We were not able to find any commercially available rat strain with proven “ouabain sensitivity” to test whether telemetric monitoring could confirm the hypertensive effect of ouabain.

The cause for the conflicting reports on the hypertensive effect of ouabain is not clear; the spectrum of possible reasons is wide and includes inaccurate arterial pressure measurements, differences in ouabain dose, method and duration of application, species, strain and age of the experimental animals, contamination of chow with cardiotonic steroids of plant origin, and the possibility of the existence of yet unknown traits that might promote or prevent ouabain-induced hypertension.

Most studies reporting ouabain-induced hypertension used tail-cuff blood pressure devices, but there is general agreement that this method is not suitable for "ruling out intermittent or subtle forms of hypertension” and “making inferences about blood pressure in non-stressed, unrestrained animals” [6]. Moreover, in many cases tail-cuff blood pressure measurement, procedures are described so superficially that assessment of the quality of the results is virtually impossible.

Currently, there is no commercially available method for the estimation of ouabain in biological tissues that provides precise and reliable results over the range of 0.01–100 nmol ouabain/L. A recent report even claims that it is not possible to detect ouabain in human plasma with ultrasensitive mass spectrometry [13]. Thus, we were not able to directly confirm elevation of the plasma levels of ouabain in our treated animals. However, we used matrix-driven delivery pellets (Innovative Research of America, Sarasota, Fl) for ouabain application; these pellets permit a non-stressful, 3-month long parenteral drug application. These pellets have proven effectiveness (www.innovrsrch.com) and have been employed in studies that have reported arterial pressure elevation associated with ouabain administration (Table S1). We directly confirmed the presence of ouabain in the pellets that we implanted and demonstrated the absence of digoxin (Figures S4–S5). Contamination with digoxin could be important because digoxin can block the hypertensive action of ouabain [14]. Rat chow can also be contaminated with cardiac glycosides of plant origin [15]. The detection limit for ouabain (3.4 nmol/ml) for the LC/MS method available at our institution was not sufficient to assess the plasma levels in rats, but analysis of the rat chow did not show ouabain or digoxin (Figure S4–S5). Thus, the concentrations of these cardiac glycosides, if present, were less than 70 nmol/g of chow.

A potentially important, but currently neglected, confounding factor could be the age of the ouabain-treated rats. In most studies, ouabain administration started at an approximate age of 6–7 weeks (Table S1–S3). At this age, arterial pressure is not stable in rats and continues to increase at a rate of 2–3 mmHg/week [9], [16], [17]. It is possible that a “time window” exists when blood pressure and its regulatory mechanisms are not fully developed and can be modified by ouabain. Rapid developmental changes in ouabain metabolism or distribution space could also be important. Because the implantation of transmitters requires both a body weight of at least ca. 200 g and a long recovery period afterwards, our rats were substantially older (21–23 weeks old) when ouabain administration began.

### Interaction of ouabain with the autonomic nervous system showed parasympathomimetic but not sympathomimetic effects

Parasympathomimetic properties of ouabain have been suggested based on its negative chronotropic [18]–[19] and dromotropic [20] effects and on its ability to stimulate acetylcholine release [21] and sensitize the baroreflex [18], [22], [23]. The heart rate in hypertensive patients with high plasma ouabain was lower than that in patients with low ouabain plasma levels [24]. It was also hypothesized that vagal stimulation could be responsible for the delay in arterial pressure elevation during chronic ouabain administration [25]. In our study, the time and frequency domain measures, the transverse axis of Poincare's plot and the Lempel-Ziv entropy of the heart rate variability were all gradually elevated during ouabain treatment, suggesting an increase in the cardiac vagal drive [26]–[28]. However, the changes in heart rate variability were significantly different only in within-group (repeated measures) testing; the between-group differences in heart rate variability, i.e., between the untreated and the ouabain-treated rats, were not significant. This finding is not surprising, as heart rate variability differs greatly between individuals (rats or humans); thus, inter-individual (between group) differences are often not significant, even if an intra-individual analysis of repeated measurements on the same subject produce statistically significant results.

The “neuromodulatory hypothesis” of ouabain action proposes that a higher brain level of ouabain activates the sympathetic system through angiotensinergic pathways and causes hypertension [4]. Although we used several different methods, i.e., blood pressure variability, pre-ejection time, ganglionic blockade, restraint stress, and urine excretion of catecholamines, no signs of sympathetic overactivity were observed.

### Missing indication of vascular pro-hypertensive remodeling in ouabain-treated animals

Proteins, particularly the Na^+^/Ca^2+^ exchanger, that regulate calcium fluxes in vascular smooth muscle cells must be upregulated before ouabain-induced hypertension can develop [2]. Consistently, during pregnancy, which is an ouabain-resistant condition with high plasma levels of ouabain and normal or reduced arterial pressure, Na^+^/Ca^2+^ exchanger is downregulated [29]. At the mRNA level, we found upregulation of the Na^+^/Ca^2+^ exchanger in the aorta but not in the mesenteric (resistance) arteries, which may explain the normal blood pressure values despite ouabain administration in our experiment.

Ouabain affects the Na^+^/Ca^2+^ exchanger indirectly through the α2 isoform of Na^+^/K^+^-ATPase. Transgenic mice with overexpression of the Na^+^/K^+^-ATPase α2 isoform were hypotensive [30], and mice with downregulation of the Na^+^/K^+^-ATPase α2 isoform had increased arterial blood pressure [31]. However, when the α2 isoform of Na^+^/K^+^-ATPase was downregulated only in the vasculature, the arterial blood pressure remained unchanged [32]. Nevertheless, the Na^+^/K^+^-ATPase α2 isoform is required for ouabain-induced hypertension because ouabain does not elevate arterial blood pressure in transgenic mice expressing the ouabain-resistant Na^+^/K^+^-ATPase α2 isoform [33]. In the mesenteric arteries in rats with ouabain-induced hypertension, the Na^+^/K^+^-ATPase α2 isoform was either increased [34] or unchanged [35]–[36]. In our rats, which were resistant to the pressor effects of ouabain, Na^+^/K^+^-ATPase α2 isoform mRNA was downregulated by 50% in the mesenteric arteries and by 66% in the aorta. This finding is consistent with reports of reduced Na^+^/K^+^-ATPase activity in ouabain-treated animals [36].

### Ouabain-treated rats did not respond to increased salt intake with arterial blood pressure elevations

Hypotheses that explain the hypertensive response to a long-term administration of ouabain postulate sympathetic activation and endothelial damage as important mechanisms [2]. Both of these alterations could interfere with the physiological response to increased salt intake and enhance the salt sensitivity of blood pressure regulation. In our experiment, ouabain did not modify the cardiovascular responses to high salt intake. The arterial blood pressure remained stable in both the control and ouabain-treated rats when the salt content in the chow changed from a very low (0.08%) to a very high concentration (8%). The autonomic nervous system response to the short-term increase in salt intake, i.e., the suppression of sympathetic activity and elevation of parasympathetic activity, also did not differ between the control and ouabain-treated rats. Augmentation of urine nitrite excretion during high salt intake is an argument against a significant renal/endothelial impairment during ouabain treatment. Although increased salt intake may stimulate the release of brain ouabain, activate the neuromodulatory pathway, and elevate the arterial pressure in salt-sensitive animals [2], [4], our data suggest that the opposite is not true; the administration of ouabain does not cause salt sensitivity.

### Increases in the plasma level of CGRP could oppose the vasoconstrictory effect of ouabain

The administration of ouabain at physiologically relevant doses never immediately increased arterial blood pressures. A delay of a minimum of one week, more often, 2–5 weeks, between the start of ouabain application and a significant arterial pressure elevation was invariably observed (Tables S1, S3). The absence of an acute hypertensive effect and the delayed onset of an arterial blood pressure increase imply that either ouabain does not have a direct vasoconstrictory effect or that this effect is offset by some vasodilatory actions, e.g., enhanced endothelial function [37], [38]–[40] or increased levels of regulator of G-protein signaling 2 protein [41]. Ouabain also stimulated the release of acetylcholine [42]–[43] and natriuretic peptides [44]. It has been proposed that ouabain-induced endothelial dysfunction could represent a crucial step in the evolution of ouabain-induced hypertension [39]. We confirmed the downregulation of nitric oxide synthase 1 and 3 gene expression in the aorta, which is a standard response to high salt intake; however, the unchanged expression of nitric oxide synthase genes in the mesenteric arteries and the normal plasma levels of cGMP suggest that there was no significant widespread damaging effect of ouabain on the endothelium. Inducible nitric oxide synthase mRNA was not detectable; this finding argues against the activation of vascular inflammation by ouabain proposed by Wenceslau and colleagues [45]. Endothelial dysfunction in ouabain-treated hypertensive rats was associated with elevated expression of cyclooxygenase 2 and increased production of vasoconstrictory prostanoids [45]–[47]. We did not find any significant changes in the mRNA expressions of cyclooxygenase 1 or 2 that were related to ouabain treatment.

CGRP is a vasodilatory neuropeptide released from the capsaicin-sensitive sensory nerve endings that innervate the cardiovascular system [48]. Both clinical studies and animal models of hypertension indicate that changes in the synthesis of, release of and/or vascular sensitivity to CGRP may have either a causative or a compensatory and protective role in the development of hypertension [49].

The increase in CGRP plasma levels in the animals treated with 324 µg ouabain/kg/day could suggest that the hypertensive effect of ouabain was offset by CGRP action. Assuming a dose-dependent effect of ouabain on CGRP secretion, the lower ouabain dose was most likely not high enough to increase the CGRP plasma levels. It is possible that due to a dense perivascular network of nervous fibers that contain and release CGRP [50], even the small amount of CGRP that did not spill over into the plasma could counteract a lower-dose ouabain vasoconstrictory effect. CGRP could also act as an endogenous antagonist of ouabain and activate Na^+^/K^+^-ATPase [51]. CGRP could delay the neurogenic vascular inflammation [52] and endothelial dysfunction [53] that have been proposed as pathogenic changes in ouabain-induced hypertension [45], [39]. Mice with augmented expression of RAMP1 (part of the CGRP receptor) have exhibited increased vagal activity and mitigation of angiotensin II-induced hypertension [54]. Thus, augmented CGRP signaling could explain our observation of parasympathomimetic effects of ouabain application. Inhibition of the α2 and α3 isoforms of Na^+^/K^+^-ATPase that are expressed in neuronal membranes and have a high affinity for ouabain could stimulate CGRP release via partial depolarization. Simultaneously, ouabain could upregulate CGRP synthesis through the activation of the Src-kinase and phosphoinositide 3-kinase pathways. However, to determine whether the documented association between the ouabain application and the elevation of CGRP in plasma is accidental or whether the relationship between CGRP and the resistance to the hypertensive effect of ouabain is indeed causative would require further experimental investigation; such studies might examine a combined application of ouabain and CGRP blocker or might test the arterial blood pressure response to ouabain in CGRP-knockout mice.

In addition to being a strong vasodilator, CGRP also has the potential to induce changes in gene expression and autonomic nervous system activity. Thus, more information about the role of CGRP in arterial pressure regulation might shed new light on the pathogenesis of essential hypertension and the role of endogenous ouabain; this knowledge could help develop new treatment modalities.

## Conclusions

Although we used experimental conditions that should produce ouabain-dependent hypertension, we were unable to reproduce this effect. In addition, we did not observe signs of sympathetic overactivity or enhanced adrenergic responsiveness. Ouabain treatment did not lead to increased blood pressure salt sensitivity. The gene expression of the Na^+^/Ca^2+^ exchanger and the Na^+^/K^+^-ATPase α2 isoform did not increase in resistance arteries. In contrast, ouabain administration was associated with parasympathomimetic effects and an elevated plasma level of CGRP.

It appears that ouabain elevates arterial pressure conditionally through a process that requires several days or weeks, during which the remodeling of arterial blood pressure regulatory mechanisms occurs and enables hypertension. This pro-hypertensive remodeling process may not necessarily occur, and the ouabain-“resistant” state associated with enhanced parasympathomimetic activity and elevated CGRP levels in plasma may develop.

## Supporting Information

Figure S1 **Mean arterial blood pressure changes displayed separately for each ouabain-treated rat.** Graph showing the mean blood pressure changes in each ouabain-treated rat relative to day 0.
(TIF)

Figure S2 **Ouabain effects on heart rate, pre-ejection time, respiratory rate, and body temperature**. The data points represent 12-hour means; the vertical bars denote 95% confidence intervals; n = 9 control rats, n = 10 ouabain-treated rats. Ouabain or placebo pellets were implanted at days 1 and 41. Between days 20–40 and 60–80, pharmacological, salt sensitivity, stress and tail-cuff simulation testing were performed. Data were analyzed with MANOVA for repeated measures and the multivariate Wilk's test (between-within design; 2 levels of main effect “group” x 2 levels of main effect “illumination” x 16 levels of main effect “time/ouabain treatment”). F, F-test value, subscripts are the degrees of freedom; P, probability. * P<0.05 when compared with the corresponding data from day 0 (Spjotvoll-Stolline post-hoc test). *Heart rate*: main effect “group,” i.e., control vs. ouabain group: F_1,17_ = 0.47, P = 0.51; main effect “time”: F_15,3_ = 10.04, P = 0.041; main effect “illumination”: F_1,17_ = 451, P<10^−6^; no significant interactions between the main effects were present. *Pre-ejection time*: main effect “group”: F_1,17_ = 1.72, P = 0.201; main effect “time”: F_15,3_ = 17.81, P = 0.018; main effect “illumination”: F_1,17_ = 229, P<10^−6^; no significant interactions between the main effects were present. *Respiratory rate*: main effect “group”: F_1,17_ = 0.43, P = 0.529; main effect “time”: F_15,3_ = 6.52, P = 0.074; main effect “illumination”: F_1,17_ = 122, P<10^−6^; no significant interactions between the main effects were present. *Body temperature*: main effect “group”: F_1,17_ = 0.7, P = 0.263; main effect “time”: F_15,3_ = 13.35, P = 0.025; main effect “illumination”: F_1,17_ = 841, P<10^−6^; no significant interactions between the main effects were present.
(TIF)

Figure S3 **Hemodynamic response to restraint stress.** The vertical bars denote 95% confidence intervals; n = 9 control rats; n = 10 ouabain-treated rats. Day 0, stress test performed before the implantation of ouabain pellets; Day 20, rats were treated for 26–30 days with 63 µg ouabain/kg/day, Day 60, rats were treated for 40 days with 63 µg ouabain/kg/day and then for 26–30 days with 324 µg ouabain/kg/day. LF SP, low-frequency variability of systolic pressure; HF RRI, high-frequency variability of RR-intervals on ECG; lnAUC, natural logarithm of the area under the curve; Δ, change from the averaged values during the 10-min period before the restraint. Data were analyzed with repeated measures MANOVA and the multivariate Wilk's test in a 2 (group) x 3 (time/ouabain treatment) x 2 (salt intake) between-within design. MANOVA results are summarized in Tables S10–S11.
(TIF)

Figure S4 **LC/MS values for ouabain and digoxin standards.** Screenshot of LC/MS Quatro LC (Micromass, UK). The standard solution of ouabain exhibits a parent mass-to-charge ratio of m/z = 584.66 (A), whereas the digoxin standard shows a m/z = 781.24 (B).
(TIF)

Figure S5 **LC/MS analysis of ouabain pellets and rats chow analysis.** Screenshot of LC/MS Quatro LC (Micromass, UK). The pellet extract exhibits only a single parent mass-to-charge ratio of m/z = 584.66, which corresponds to ouabain (A). The parent mass-to-charge ratio indicating the presence of digoxin (m/z = 781.24) was absent in the pellet extract. The diet extract was also measured under the same conditions, and no parent mass-to-charge ratios for ouabain or digoxin were detected (B).
(TIF)

Table S1 **Reports in favor of a hypertensive effect of exogenous ouabain.**
(PDF)

Table S2 **Reports against a hypertensive effect of exogenous ouabain.**
(PDF)

Table S3 **Studies that showed variable sensitivity to the hypertensive effects of ouabain.**
(PDF)

Table S4 **Cosinor analysis of the circadian variability of mean arterial blood pressure.**
(PDF)

Table S5 **Time- and frequency-domain indices of heart rate variability.**
(PDF)

Table S6 **Complexity and self-similarity of heart rate variability.**
(PDF)

Table S7 **Baroreflex sensitivity.**
(PDF)

Table S8 **Time- and frequency-domain indices of systolic pressure variability.**
(PDF)

Table S9 **Complexity and self-similarity of systolic pressure variability.**
(PDF)

Table S10 **Hemodynamic responses to restraint stress (MANOVA results).**
(PDF)

Table S11 **Blood pressure and heart rate variability responses to restraint stress (MANOVA results).**
(PDF)

Methods S1 **Detailed description of the experimental procedures.**
(PDF)
