# Supplementary material for: Wistar Rats Resistant to the Hypertensive Effects of Ouabain Exhibit Enhanced Cardiac Vagal Activity and Elevated Plasma Levels of Calcitonin Gene-Related Peptide
Source: PLoS One. 2014 Oct 3;9(10):e108909. doi: 10.1371/journal.pone.0108909 (PMC4184851; doi:10.1371/journal.pone.0108909)
Supplement: Table S1 — Reports in favor of a hypertensive effect of exogenous ouabain. (PDF) [file pone.0108909.s006.pdf]

Table S1. Reports in favor of a hypertensive effect of exogenous ouabain

Study	Species/ Strain	Age/weight Sample size	Method of BP measurement	Dose	Duration	Route of administration	Cardiovascular effects	Publications by same group
Yuan et al. [1]	Wistar rats Normal RRM: 25,60,70%	350-400 g, n=5-7	Tail-cuff Femoral artery cath.*	17, 28, 34 µg/kg/day	6-8 weeks	i.p daily	↑MAP in 70>60>25%>2 Kidney by ~20 to 30 mm Hg (2 weeks) ↑or ↔ in HR	[2-4, 5**, 6, 7**, 8-11]
Manunta et al. [2]	SD rats	7-8 weeks 200-230 g n=8	Tail-cuff (photoelectric)	3,10, 15, 30 µg/kg/day	5 weeks	s.c osmotic mini-pump 12 µl/day for 14 days	↑ SP, MAP dose dependently by ~15 to 40 mm Hg (2 weeks) ↔ HR	
Pulina et al. [3]	SD rats	300 g, n=7-8	Tail-cuff (photoelectric)	25 µg/day	5 weeks	s.c 1.5 mg/pellet/60day	↑ SP by ~30 mm Hg (2 weeks)	
Huang et al. [12]	Wistar rats	200-250 g n=7-9	Chronic carotid cath.**	10, 25, 75µg/day	14 days	i.v osmotic mini-pump s.c 0.5 mg/pellet/21days	↑MAP by ~27 mm Hg (10 days) ↑ HR by ~40 beats/min	[13**, 14**, 15**]
Kurashina et al. [16]	SD rats	180-200 g n=9	Carotid and femoral cath.*	28 µg/kg/day	6 weeks	i.p daily	↑ MAP by ~35 mm Hg	
Wang et al. [17]	SD rats	7-11 weeks 200-250 g n=24	Hind leg-cuff Aortic cath.*	24 µg/kg/day	6 weeks	i.p daily	↑ SP by ~27 mm Hg (2 weeks)	[18]
Rossoni et al. [19]	Wistar rats	6 weeks n=24-35	Tail-cuff	8, 25 µg/day	5 weeks	s.c 0.5 mg/pellet/60days	↑ SP by ~30 mm Hg (1 week)	[20-22, 23**, 24-27, 28*, 29-30]
Padilha et al. [20]	Wistar rats	8-12 weeks n=6	Carotid cath.*	25 µg/kg/day	3, 7, 15, 30 days	s.c daily ouabain diluted in soy oil	↑ SP by ~20 mm Hg (2 weeks) ↑ DP by ~15 mm Hg (1 week) ↑ HR at day 15 but ↔ at day30	
Xavier et al. [21]	SHR	6 weeks n=10-11	Carotid cath.**	8 µg/day	5 weeks	s.c 0.5 mg/pellet/60days	↑ SP by ~22 mm Hg in SHR ↑ DP by ~20 mm Hg in SHR	

Table S1 (continued). Reports in favor of a hypertensive effect of exogenous ouabain

Study	Species/ strain	Age/weight Sample size	Method of BP measurement	Dose	Duration	Route of administration	Cardiovascular effects	Publications by same group
Di et al. [31]	SD rats	250-300 g n=6	Femoral cath.*	7,14, 28 µg/kg/day	4 weeks	s.c osmotic mini-pump	↑ MAP in 14 and 28 µg/kg/day by ~40-70 mm Hg	
Dostanic et al. [32]	Mice	n=5	Tail-cuff	30, 100, 300 µg/kg/day	5 weeks	i.p daily	↑ SP in 300 µg/kg/day only by ~20 mm Hg (5 days) ↔ HR	
Holthouser et al. [33]	SD rats	200-250 g n=8	Carotid cath.*	1 µg/kg/day	8 days	i.p daily	↑ SP and DP by ~21 mm Hg ↑ MAP by ~19 mm Hg (8 days) ↔ HR	
Silva et al. [34]	WKY rats	5 weeks 45weeks n=4-6	Tail-cuff (photoelectric)	8 µg/day	7 weeks	s.c 0.5 mg/pellet/60days	↑ SP by~25 mm Hg (3-5 weeks)	
Jacobs et al. [35]	SD rats Non-pregnant	200-225 g n=8	Tail-cuff	21 µg/kg/day	16 days	s.c osmotic mini-pump	↑ SP by ~24 mm Hg	
Pulgar et al. [36]	SD rats	12 weeks n=5-6	Tail-cuff	25 µg/day	8 weeks	s.c 1.5 mg/pellet/60days	↑ SP by ~40 mm Hg (7weeks)	
Zhao et al. [37]	SD rats	6-8 weeks n=20	Tail-cuff	28 µg/kg	6 weeks	i.p daily	↑ SP by ~20 mm Hg (5 weeks)	[38]

SD, Sprague Dawley; WKY, Wistar Kyoto; SHR, Spontaneously hypertensive rats; RRM, reduced renal mass; cath., catheterization; i.p, intraperitoneal; i.v, intravenous; s.c, subcutaneous; SP, systolic pressure; DP, diastolic pressure; MAP, mean arterial pressure; HR, heart rate. *, blood pressure measured under anesthesia; **, 4-48 hours of recovery before blood pressure measurement. Numbers between brackets represent the time required for hypertension to develop.

References

1. Yuan CM, Manunta P, Hamlyn JM, Chen S., Bohen E, et al. (1993) Long-term ouabain administration produces hypertension in rats. Hypertension 22: 178-187.

2. Manunta P, Rogowski AC, Hamilton BP, Hamlyn JM (1994) Ouabain-induced hypertension in the rat: relationships among plasma and tissue ouabain and blood pressure. J Hypertens12: 549-560.

3. Pulina MV, Zulian A, Berra-Romani R, Beskina O, Mazzocco-Spezzia A, et al. (2010) Upregulation of Na+ and Ca2+ transporters in arterial smooth muscle from ouabain-induced hypertensive rats. Am J Physiol Heart Circ Physiol 298: H263-H274.

4. Pamnani MB, Chen S, Yuan CM, Haddy FJ et al. (1994) Chronic blood pressure effects of bufalin, a sodium-potassium ATPase inhibitor, in rats. Hypertension 23: I106-I109.

5. Manunta P, Hamilton J, Rogowski AC, Hamilton BP, Hamlyn JM (2000) Chronic hypertension induced by ouabain but not digoxin in the rat: antihypertensive effect of digoxin and digitoxin. Hypertens Res 23Suppl: S77-S85.

6. Kimura K, Manunta P, Hamilton BP, Hamlyn JM (2000) Different effects of in vivo ouabain and digoxin on renal artery function and blood pressure in the rat. Hypertens Res 23Suppl: S67-S76.

7. Manunta P, Hamilton BP, Hamlyn JM (2001) Structure-activity relationships for the hypertensinogenic activity of ouabain: role of the sugar and lactone ring. Hypertension 37: 472-477.

8. Iwamoto T, Kita S, Zhang J, Blaustein MP, Arai Y, et al. (2004) Salt-sensitive hypertension is triggered by Ca2+ entry via Na+/Ca2+ exchanger type-1 in vascular smooth muscle. Nat Med 10: 1193-1199.

9. Cao C, Payne K, Lee-Kwon W, Zhang Z, Lim SW, et al. (2009) Chronic ouabain treatment induces vasa recta endothelial dysfunction in the rat. Am J Physiol Renal Physiol 296: F98-F106.

10. Zhang J, Hamlyn JM, Karashima E, Raina H, Mauban JR, et al. (2009) Low-dose ouabain constricts small arteries from ouabain-hypertensive rats: implications for sustained elevation of vascular resistance. Am J Physiol Heart Circ Physiol 297: H1140-H1150.

11. Zulian A, Linde CI, Pulina MV, Baryshnikov SG, Papparella I, et al. (2013) Activation of c-SRC underlies the differential effects of ouabain and digoxin on Ca(2+) signaling in arterial smooth muscle cells. Am J Physiol Cell Physiol 304(4): C324-33.

12. Huang BS, Huang X, Harmsen E, Leenen FH (1994) Chronic central versus peripheral ouabain, blood pressure, and sympathetic activity in rats. Hypertension 23: 1087-1090.

13. Huang BS, Kudlac M, Kumarathasan R, Leenen FH (1999) Digoxin prevents ouabain and high salt intake-induced hypertension in rats with sinoaortic denervation. Hypertension 34: 733-738.

14. Veerasingham SJ, Vahid-Ansari F, Leenen FH (2000) Neuronal Fos-like immunoreactivity in ouabain-induced hypertension. Brain Res 876: 17-21.

15. Cheung WJ, Kent MA, El-Shahat E, Wang H, Tan J, et al. (2006) Central and peripheral renin-angiotensin systems in ouabain-induced hypertension. Am J Physiol Heart Circ Physiol 291: H624-H630.

16. Kurashina T, Kirchner KA, Granger JP, Patel AR, et al. (1996) Chronic sodium-potassium-ATPase inhibition with ouabain impairs renal haemodynamics and pressure natriuresis in the rat. Clin Sci (Lond) 91: 497-502.

17. Wang H, Lu Z, Yuan W (1997) Comparative study of the effects of ouabain and digoxin on blood pressure of rats. Chin Med J (Engl) 110: 911-914.

18. Wang H, Yuan WQ, Lu ZR (2000) Differential regulation of the sodium pump alpha-subunit isoform gene by ouabain and digoxin in tissues of rats. Hypertens Res 23: SupplS55-S60.

19. Rossoni LV, Salaices M, Marin J, Vassallo DV, Alonso MJ (2002) Alterations in phenylephrine-induced contractions and the vascular expression of Na+,K+-ATPase in ouabain-induced hypertension. Br J Pharmacol 135: 771-781.

20. Padilha AS, Moreira CM, Meira EF, Siman FD, Stefanon I, et al. (2008) Chronic ouabain treatment enhances cardiac myosin ATPase activity in rats. Clin Exp Pharmacol Physiol 35: 801-806.

21. Xavier FE, Davel AP, Fukuda LE, Rossoni LV (2009) Chronic ouabain treatment exacerbates blood pressure elevation in spontaneously hypertensive rats: the role of vascular mechanisms. J Hypertens 27: 1233-1242.

22. Xavier FE, Rossoni LV, Alonso MJ, Balfagon G, Vassallo DV, et al. (2004) Ouabain-induced hypertension alters the participation of endothelial factors in alpha-adrenergic responses differently in rat resistance and conductance mesenteric arteries. Br J Pharmacol 143: 215-225.

23. Xavier FE, Salaices M, Márquez-Rodas I, Alonso MJ, Rossoni LV, et al. (2004) Neurogenic nitric oxide release increases in mesenteric arteries from ouabain hypertensive rats. J Hypertens 22: 949-957.

24. Xavier FE, Yogi A, Callera GE, Tostes RC, Alvarez Y, et al. (2004) Contribution of the endothelin and renin-angiotensin systems to the vascular changes in rats chronically treated with ouabain. Br J Pharmacol 143: 794-802.

25. Rossoni LV, Salaices M, Miguel M, Briones AM, Barker LA, et al. (2002) Ouabain-induced hypertension is accompanied by increases in endothelial vasodilator factors. Am J Physiol Heart Circ Physiol 283: H2110-H2118.

26. Briones AM, Xavier FE, Arribas SM, González MC, Rossoni LV, et al. (2006) Alterations in structure and mechanics of resistance arteries from ouabain-induced hypertensive rats. Am J Physiol Heart Circ Physiol 291: H193-H201.

27. Hernanz R, Briones AM, Martín A, Beltrán AE, Tejerina T, et al. (2008) Ouabain treatment increases nitric oxide bioavailability and decreases superoxide anion production in cerebral vessels. J Hypertens 26: 1944-1954.

28. Padilha AS, Pecanha FM, Vassallo DV, Alonso MJ, Salaices M (2008) Ouabain treatment changes the role of endothelial factors in rat resistance arteries. Eur J Pharmacol 600: 110-116.

29. Briones AM, Padilha AS, Cogolludo AL, Alonso MJ, Vassallo DV, et al. (2009) Activation of BKCa channels by nitric oxide prevents coronary artery endothelial dysfunction in ouabain-induced hypertensive rats. J Hypertens 27: 83-91.

30. Wenceslau CF, Davel AP, Xavier FE, Rossoni LV (2011) Long-term ouabain treatment impairs vascular function in resistance arteries. J Vasc Res 48: 316-326.

31. Di FC, Filippelli A, Rinaldi B, Piegari E, Esposito F, et al. (2003) Chronic peripheral ouabain treatment affects the brain endothelin system of rats. J Hypertens 21: 747-753.

32. Dostanic I, Paul RJ, Lorenz JN, Theriault S, Van Huysse JW, et al. (2005) The alpha2-isoform of Na-K-ATPase mediates ouabain-induced hypertension in mice and increased vascular contractility in vitro. Am J Physiol Heart Circ Physiol 288: H477-H485.

33. Holthouser KA, Mandal A, Merchant ML, Schelling JR, Delamere NA, et al. (2010) Ouabain stimulates Na-K-ATPase through a sodium/hydrogen exchanger-1 (NHE-1)-dependent mechanism in human kidney proximal tubule cells. Am J Physiol Renal Physiol 299: F77-F90.

34. Silva E, Serrao MP, Soares-da-Silva P (2011) Age-dependent effect of ouabain on renal Na+,K+-ATPase. Life Sci 88: 719-724.

35. Jacobs BE, Liu Y, Pulina MV, Golovina VA, Hamlyn JM (2012) Normal pregnancy: mechanisms underlying the paradox of a ouabain-resistant state with elevated endogenous ouabain, suppressed arterial sodium calcium exchange, and low blood pressure. Am J Physiol Heart Circ Physiol 302: H1317-H1329.

36. Pulgar VM, Jeffers AB, Rashad HM, Diz DI, Aileru AA (2013) Increased constrictor tone induced by ouabain treatment in rats. J Cardiovasc Pharmacol 62(2): 174-83.

37. Zhao SH, Gao HQ, Ji X, Wang Y, Liu XJ, et al. (2013) Effect of ouabain on myocardial ultrastructure and cytoskeleton during the development of ventricular hypertrophy. Heart Vessels 28(1): 101-13.

38. Liu X, Qiu J, Zhao S, You B, Ji X, et al. (2012) Grape seed proanthocyanidin extract alleviates ouabain-induced vascular remodeling through regulation of endothelial function. Mol Med Rep 6(5): 949-54.
