# Supplementary material for: Wistar Rats Resistant to the Hypertensive Effects of Ouabain Exhibit Enhanced Cardiac Vagal Activity and Elevated Plasma Levels of Calcitonin Gene-Related Peptide
Source: PLoS One. 2014 Oct 3;9(10):e108909. doi: 10.1371/journal.pone.0108909 (PMC4184851; doi:10.1371/journal.pone.0108909)
Supplement: Table S2 — Reports against a hypertensive effect of exogenous ouabain. (PDF) [file pone.0108909.s007.pdf]

Table S2. Reports against a hypertensive effect of exogenous ouabain

Study	Species/strain	Age/weight Sample size	Method of BP measurement	Dose	Duration	Route of administration	Cardiovascular effects
Nirasawa et al. [1]	SD rats,1 kidney	250-300 g n=5-6	Tail-cuff	1, 5, 10 mg/kg/day	26 days	i.p daily	↔ SP
Yasujima et al. [2]	SD rats, 2 kidneys, 1 kidney	180-280 g n=7	Tail-cuff	1.2 mg/kg/day	6 days	i.v osmotic mini-pump	↔ SP
Sekihara et al. [3]	SD rats,1 kidney	130-150 g n=4-6	Tail-cuff	143, 285 μg/day	6 weeks	1 or 2 mg once a week in sesame oil	↔ SP
Li et al. [4]	SD rats	270-350 g n=14	Tail-cuff, Carotid cath.*	10, 100 μg/kg/day	4 weeks	i.p osmotic mini-pump	↔ SP
Nelissen et al. [5]	Wistar rats	370-400 g n=6-8	Abdominal aorta cath.**	10 μg/kg/day	2 weeks	s.c osmotic mini-pump	↔ MAP, ↔ HR
Wang et al. [6]	Long-Evans rats 2kidneys, 1 kidney	350-450 g n=5	Chronic abdominal aorta cath.***	30 μg/kg/day	4 weeks	i.v syringe pump	↔ MAP
Cargnelli et al. [7]	SD rats	350-400 g n=20	Tail-cuff	50 μg/kg/day	4 weeks	s.c osmotic mini-pump	↔ SP
Neri et al. [8]	WKY rats	226±10 g n=10	Tail-cuff	100 μg/kg/day	4 months	s.c osmotic mini-pump	↔ SP
Xavier et al. [9]	WKY rats	6 weeks n=6-7	Carotid cath.**	8 μg/day	5 weeks	s.c 0.5 mg/pellet/60days	↔ SP
Jacobs et al. [10]	SD rats pregnant	200-225 g n=11	Tail-cuff	21 μg/kg/day	16 days	s.c osmotic mini-pump	↔ SP
Pidgeon et al. [11]	Sheep	41-52 kg n=8	Chronic carotid cath.***	0.25 mg/day, higher doses are lethal	22 days	i.v infusion	↔ MAP, ↔ HR

SD, Sprague Dawley; WKY, Wistar Kyoto; i.p, intraperitoneal; i.v, intravenous; s.c, subcutaneous; SP, systolic pressure; MAP, mean arterial pressure; HR, heart rate; cath, catheterization. *, blood pressure measured under anesthesia; **, 4-48 hours of recovery before blood pressure measurement; ***, 6-10 days of recovery before blood pressure measurement.

References

1. Nirasawa Y, Temma K, Fink GD, Akera T (1985) In ability of Na+,K+-ATPase inhibitor to cause hypertension in sodium-loaded or deoxycorticosterone-treated one kidney rats. Life Sci 37: 767-774.

2. Yasujima M, Abe K, Tanno M, Kohzuki M, Kasai Y, et al. (1986) Effects of ouabain on blood pressure regulation in rats. J Hypertens 4: 597-601.

3. Sekihara H, Yazaki Y, Kojima T (1992) Ouabain as an amplifier of mineralocorticoid-induced hypertension. Endocrinology 131: 3077-3082.

4. Li M, Martin A, Wen C, Turner SW, Lewis LK, et al. (1995) Long-term ouabain administration does not alter blood pressure in conscious Sprague-Dawley rats. Clin Exp Pharmacol Physiol 22: 919-923.

5. Nelissen-Vrancken HJ, Wang JF, Struijker Boudier HA, Schoemaker RG, Smits JF (1997) Ouabain improves cardiac function in vivo in rats with heart failure after chronic but not acute treatment. Naunyn Schmiedebergs Arch Pharmacol 356: 203-209.

6. Wang J, Tempini A, Schnyder B, Montani JP (1999) Regulation of blood pressure during long-term ouabain infusion in Long-Evans rats. Am J Hypertens 12: 423-426.

7. Cargnelli G, Trevisi L, Debetto P, Luciani S, Bova S (2000) Effect of long-term ouabain treatment on contractile responses of rat aortae. J Cardiovasc Pharmacol 35: 538-542.

8. Neri G, De TR, Tortorella C, Rebuffat P, Bova S, et al. (2006) Ouabain chronic infusion enhances the growth and steroidogenic capacity of rat adrenal zona glomerulosa: the possible involvement of the endothelin system. Int J Mol Med 18: 315-319.

9. Xavier FE, Davel AP, Fukuda LE, Rossoni LV (2009) Chronic ouabain treatment exacerbates blood pressure elevation in spontaneously hypertensive rats: the role of vascular mechanisms. J Hypertens 27: 1233-1242.

10. Jacobs BE, Liu Y, Pulina MV, Golovina VA, Hamlyn JM (2012) Normal pregnancy: mechanisms underlying the paradox of a ouabain-resistant state with elevated endogenous ouabain, suppressed arterial sodium calcium exchange, and low blood pressure. Am J Physiol Heart Circ Physiol 302: H1317-H1329.

11. Pidgeon GB, Richards AM, Nicholls MG, Charles CJ, Rademaker MT, et al. (1996) Chronic ouabain infusion does not cause hypertension in sheep. Am J Physiol 270: E386-E392.
