# Supplementary material for: Wistar Rats Resistant to the Hypertensive Effects of Ouabain Exhibit Enhanced Cardiac Vagal Activity and Elevated Plasma Levels of Calcitonin Gene-Related Peptide
Source: PLoS One. 2014 Oct 3;9(10):e108909. doi: 10.1371/journal.pone.0108909 (PMC4184851; doi:10.1371/journal.pone.0108909)
Supplement: Table S3 — Studies that showed variable sensitivity to the hypertensive effects of ouabain. (PDF) [file pone.0108909.s008.pdf]

Table S3. Studies that showed variable sensitivity to the hypertensive effects of ouabain

Study	Species/ strain	Age/weight Sample size	Method of BP measurement	Dose	Duration	Route of administration	Cardiovascular effects	Publications by same group
Manunta et al. [1]	SD rats	7-8 weeks<br>200-230 g<br>n=8	Tail-cuff	30 µg/kg/day	5 weeks	s.c osmotic mini-pump	↑ SP~30-50 mm Hg in 7 rats ~88%, (2 weeks), ↔SP in 1 rat ~15%	
Ferrari et al. [2]	SD rats	6-7 weeks<br>150-180 g<br>n=40	Tail-cuff	50 µg/kg/day	8 weeks	s.c osmotic mini-pump	↑ SP ~20-25 mm Hg in ~70-80% (3-4 weeks), ↔ HR	
Aileru et al. [3]	SD rats	200-250 g<br>n=40 males<br>n=40 females	Tail-cuff (Photoelectric)	30 µg/kg/day	5 weeks	s.c osmotic mini-pump	↑ SP ~14 mm Hg in~92% (BOS), (1 week), ↔ SP in ~8% (BOR)	
Tian et al. [4]	SD rats	150-200 g<br>n=10-20	Tail-cuff (7 days of pre-training)	28 µg/kg/day	6 weeks	i.p daily	↑ SP ~ 40 mm Hg in ~65% (2 weeks)	
Ren et al. [5]	SD rats	180-250 g<br>n=10-20	Tail-cuff	20 µg/kg/day	8 weeks	i.p daily	↑ SP ~18 mm Hg in ~83% (OS), (6 weeks), ↔ SP in ~17% (OR)	[6-8]

SD, Sprague Dawley; i.p, intraperitoneal; s.c, subcutaneous; SP, systolic pressure; HR, heart rate; BOS, Baltimore ouabain sensitive strain; BOR, Baltimore ouabain resistant strain; OS, ouabain sensitive; OR, ouabain resistant. Numbers between brackets represent the time required for hypertension to develop.

References

1. Manunta P, Rogowski AC, Hamilton BP, Hamlyn JM (1994) Ouabain-induced hypertension in the rat: relationships among plasma and tissue ouabain and blood pressure. J Hypertens 12: 549-560.

2. Ferrari P, Torielli L, Ferrandi M, Padoani G, Duzzi L, et al. (1998) PST2238: a new antihypertensive compound that antagonizes the long-term pressor effect of ouabain. J Pharmacol Exp Ther 285: 83-94.

3. Aileru AA, De AA, Hamlyn JM, Manunta P, Shah JR, et al. (2001) Synaptic plasticity in sympathetic ganglia from acquired and inherited forms of ouabain-dependent hypertension. Am J Physiol Regul Integr Comp Physiol 281: R635-R644.

4. Tian G, Dang C, Lu Z (2001) The change and significance of the Na+-K+-ATPase alpha-subunit in ouabain-hypertensive rats. Hypertens Res 24: 729-734.

5. Ren Y, Zhang M, Zhang T, Huang R (2013) Effect of ouabain on myocardial remodeling in rats. Exp Ther Med 6(1): 65-70.

6. Ren YP, Huang RW, Lu ZR (2006). Ouabain at pathological concentrations might induce damage in human vascular endothelial cells. Acta Pharmacol Sin 27: 165-172.

7. Jiang X, Ren YP, Lv ZR (2007) Ouabain induces cardiac remodeling in rats independent of blood pressure. Acta Pharmacol Sin 28: 344-352.

8. Zhang Y, Yuan Z, Ge H, Ren Y (2010) Effects of long-term ouabain treatment on blood pressure, sodium excretion, and renal dopamine D(1) receptor levels in rats. J Comp Physiol B 180: 117-124.
