# Supplementary material for: Wistar Rats Resistant to the Hypertensive Effects of Ouabain Exhibit Enhanced Cardiac Vagal Activity and Elevated Plasma Levels of Calcitonin Gene-Related Peptide
Source: PLoS One. 2014 Oct 3;9(10):e108909. doi: 10.1371/journal.pone.0108909 (PMC4184851; doi:10.1371/journal.pone.0108909)
Supplement: Table S4 — Cosinor analysis of the circadian variability of mean arterial blood pressure. (PDF) [file pone.0108909.s009.pdf]

Table S4. Cosinor analysis of circadian variability of mean arterial blood pressure

	Control			Ouabain treated			Main effects			
	Ouabain 0 µg/kg			0 µg/kg	63 µg/kg	324 µg/kg	Group		Time	
	Day 0	Day 20	Day 60	Day 0	Day 20	Day 60	$F_{1,17}$	P	$F_{2,16}$	P
**Coefficient of determination (%)**	12 (9)	10 (8)	9 (4)	8 (6)	14 (9)	16 (6)	0.87	0.365	0.71	0.511
**Mean (dark period), mmHg**	115 (6)	115 (7)	115(7)	119 (6)	119 (7)	120 (7)	2.49	0.133	2.59	0.105
**Mean (light period), mmHg**	111 (6)	110 (6)	111 (6)	116 (6)	113 (5)#	114 (6)	2.93	0.105	3.91	0.042
**Maximum, mmHg**	117 (6)	116 (7)	117 (7)	120 (6)	120 (7)	121 (7)	2.43	0.137	2.43	0.119
**Acrophase, hour**	14.3 (1)	13.5 (1)	11.6 (1.3)*	12.6 (1)	12.9 (1.2)	12.1 (1)	2.44	0.137	13.86	3.10^{-4}
**Minimum, mmHg**	111 (6)	110 (6)	110 (6)	116 (6)	113 (5)#	114 (6)	2.62	0.124	3.80	0.045
**Time of minimum, hour**	2.3 (1.5)	1.4 (1)	23.6 (1.3)*	0.6 (1.5)	1.0 (1.2)	24.1 (0.3)	2.44	0.137	9.32	6.10^{-4}
**Maximum-minimum difference, mm Hg**	6 (3)	6 (3)	6 (2)	5 (3)	7 (3)	8 (2)	0.37	0.553	3.09	0.073
**Maximal slope, mmHg/h**	0.8 (0.4)	0.8 (0.4)	0.8 (0.3)	0.7 (0.4)	0.9 (0.4)	1.1 (0.3)	0.36	0.552	3.09	0.073

Values are means (standard deviation); n= 9 control rats; n = 10 ouabain treated rats. Coefficient of determination, percentage of variation in the data that is explained by the fitted model. Main effects and their interactions were tested with repeated measures MANOVA and multivariate Wilks tests for repeated measure; *P < 0.007 vs. control *Day 0*; #P < 0.004 vs. ouabain *Day 0* with Spjotvoll-Stolline post hoc test.
