# Supplementary material for: Wistar Rats Resistant to the Hypertensive Effects of Ouabain Exhibit Enhanced Cardiac Vagal Activity and Elevated Plasma Levels of Calcitonin Gene-Related Peptide
Source: PLoS One. 2014 Oct 3;9(10):e108909. doi: 10.1371/journal.pone.0108909 (PMC4184851; doi:10.1371/journal.pone.0108909)
Supplement: Table S5 — Time- and frequency-domain indices of heart rate variability. (PDF) [file pone.0108909.s010.pdf]

Table S5. Time and frequency domain indices of heart rate variability

	Control Ouabain 0 µg/kg Day 0		Control Ouabain 0 µg/kg Day 20		Control Ouabain 0 µg/kg Day 60		Ouabain treated 0 µg/kg Day 0		Ouabain treated 63 µg/kg Day 20		Ouabain treated 324 µg/kg Day 60	
	**Dark**	**Light**	**Dark**	**Light**	**Dark**	**Light**	**Dark**	**Light**	**Dark**	**Light**	**Dark**	**Light**
**RRI ms**	155 (8)	198 (10)	157(9)	207 (9)	158 (8)	194 (13)	161 (8)	197 (10)	157 (13)	203 (11)	157 (11)	199 (13)
**SD of RRI ms**	15 (3)	16 (4)	18 (3)	15 (4)	18 (4)	21 (6)	19 (4)	19 (5)	17 (3)	15 (3)	17 (3)	18 (5)
**RMSSD ms**	2.6 (0.8)	4.1 (1.7)	2.7 (0.8)	3.5 (1.1)	2.7 (0.8)	4.3 (1.4)	3.5 (1.1)	4.4 (1.7)	3.1 (0.6)	4.8 (1.4)	3.7 (1.1)	5.7 (2.7)
**TP lnAUC ms^2.s**	11.5 (0.4)	11.7 (0.5)	11.7 (0.3)	11.7 (0.5)	11.7 (0.4)	12 (0.5)	11.9 (0.4)	12 (0.4)	11.6 (0.4)	11.8 (0.3)	11.8 (0.4)	12 (0.4)
**VLF lnAUC ms^2.s**	11.4 (0.4)	11.6 (0.5)	11.6 (0.3)	11.6 (0.6)	11.6 (0.4)	11.9 (0.5)	11.8 (0.5)	11.9 (0.5)	11.5 (0.4)	11.6 (0.3)	11.6 (0.4)	11.9 (0.4)
**LF lnAUC ms^2.s**	8.7 (0.5)	9.1 (0.6)	8.8 (0.5)	8.9 (0.6)	8.7 (0.5)	9.2 (0.7)	9.1 (0.3)	9.3 (0.4)	8.9 (0.3)	9.3 (0.4)	9.0 (0.3)	9.7 (0.4)
**HF lnAUC ms^2.s**	7.5 (0.5)	8.0 (0.7)	7.6 (0.5)	7.8 (0.5)	7.6 (0.5)	8.2 (0.7)	8.0 (0.5)	8.2 (0.68)	7.8 (0.4)	8.2 (0.4)	7.9 (0.5)	8.5 (0.65)
**LF/HF**	1.43 (0.29)	1.29 (0.5)	1.41 (0.5)	1.40 (0.68)	1.41 (0.47)	1.23 (0.52)	1.61 (0.68)	1.44 (0.61)	1.61 (0.65)	1.43 (1.02)	1.52 (0.64)	1.41 (0.63)

Values are means (standard deviation); n = 9 control rats; n = 10 ouabain treated rats. RRI, time period between subsequent R-waves on the ECG; SD, standard deviation; RMSSD, square root of the mean sum of the squares of differences between adjacent RR-intervals; ln, natural logarithm; AUC, area under curve; TP, total power of heart rate variability; VLF, very low frequency power of heart rate variability; LF, low frequency power of heart rate variability; HF, high frequency power of heart rate variability. Spectral powers were determined in 2s intervals (Wigner-Ville transform) in 35 min long segments, then integrated over the whole 35min and logarithmically transformed. Data are averages of results of variability analysis in first two complete segments after 12 p.m. and 12 a.m.

(Statistical results are on next page).

Table S5. Time and frequency domain indices of heart rate variability (MANOVA results)

	Interactions								Main effects					
	3-way		Time x Group		Illumination x Group		Time x Illumination		Group		Time		Illumination	
	$F_{2,16}$	P	$F_{2,16}$	P	$F_{1,17}$	P	$F_{2,16}$	P	$F_{1,17}$	P	$F_{2,16}$	P	$F_{1,17}$	P
**RRI ms**	1.2	0.319	1.0	0.401	0.2	0.699	4.4	0.03	0.1	0.797	2.6	0.104	384	10^{-6}
**SD of RRI ms**	0.5	0.633	6.2	0.01	0.01	0.893	4.4	0.03	0.1	0.789	3.3	0.061	0.1	0.733
**RMSSD ms**	2.1	0.156	1.3	0.304	0.4	0.558	0.8	0.237	2.9	0.107	4.7	0.024	36.5	10^{-5}
**TP lnAUC ms^2.s**	0.8	0.467	2.6	0.108	0.09	0.769	2.5	0.114	0.7	0.412	2.6	0.109	8.9	0.008
**VLF lnAUC ms^2.s**	0.6	0.548	3.0	0.079	0.2	0.707	2.3	0.130	0.5	0.505	2.4	0.126	6.1	0.024
**LF lnAUC ms^2.s**	1.82	0.192	0.3	0.725	0.01	0.934	3.5	0.054	3.3	0.086	2.9	0.086	66.4	10^{-6}
**HF lnAUC ms^2.s**	1.8	0.191	0.04	0.960	0.03	0.863	2.8	0.089	2.4	0.138	3.8	0.045	45.9	3.10^{-6}
**LF/HF**	0.3	0.750	0.04	0.964	0.06	0.806	0.08	0.921	0.3	0.591	0.9	0.416	2.4	0.138

Within groups main effects and their interactions were tested with repeated measures MANOVA and multivariate Wilks test; between groups main effect “group” was tested with the univariate ANOVA (between-within design; 2 levels of main effect “group” x 2 levels of main effect “illumination” x 3 levels of main effect “time/ouabain treatment”). RRI, time period between subsequent R-waves on the ECG; SD, standard deviation; RMSSD, square root of the mean sum of the squares of differences between adjacent RR-intervals; ln, natural logarithm; AUC, area under curve; TP, total power of heart rate variability; VLF, very low frequency power of heart rate variability; LF, low frequency power of heart rate variability; HF, high frequency power of heart rate variability; F, multivariate (repeated measures factors) or univariate (between groups factor) F-test values, subscripts are degrees of freedom; P, probability.
