# Supplementary material for: Wistar Rats Resistant to the Hypertensive Effects of Ouabain Exhibit Enhanced Cardiac Vagal Activity and Elevated Plasma Levels of Calcitonin Gene-Related Peptide
Source: PLoS One. 2014 Oct 3;9(10):e108909. doi: 10.1371/journal.pone.0108909 (PMC4184851; doi:10.1371/journal.pone.0108909)
Supplement: Table S6 — Complexity and self-similarity of heart rate variability. (PDF) [file pone.0108909.s011.pdf]

Table S6. Complexity and self-similarity of heart rate variability

	Control<br>Ouabain 0 µg/kg<br>Day 0		Day 20		Day 60		Ouabain treated<br>0 µg/kg<br>Day 0		63 µg/kg<br>Day 20		324 µg/kg<br>Day 60	
	**Dark**	**Light**	**Dark**	**Light**	**Dark**	**Light**	**Dark**	**Light**	**Dark**	**Light**	**Dark**	**Light**
**Lempel-Ziv entropy**	0.23 (0.04)	0.31 (0.05)	0.19 (0.03)	0.29 (0.07)	0.20 (0.03)	0.27 (0.05)	0.22 (0.06)	0.31 (0.09)	0.23 (0.03)	0.39 (0.09)	0.24 (0.06)	0.37 (0.09)
**Shannon's entropy**	0.62 (0.03)	0.63 (0.03)	0.59 (0.03)	0.62 (0.04)	0.60 (0.03)	0.58 (0.04)	0.59 (0.06)	0.61 (0.07)	0.59 (0.06)	0.65 (0.08)	0.60 (0.08)	0.64 (0.11)
**Fractal dimension**	1.44 (0.17)	1.49 (0.17)	1.44 (0.10)	1.50 (0.19)	1.43 (0.16)	1.47 (0.18)	1.47 (0.18)	1.51 (0.19)	1.48 (0.17)	1.56 (0.21)	1.49 (0.18)	1.56 (0.21)
**Alpha S**	1.12 (0.09)	1.04 (0.14)	1.09 (0.12)	1.07 (0.19)	1.09 (0.12)	1.03 (0.13)	1.10 (0.15)	1.03 (0.16)	1.10 (0.15)	1.00 (0.21)	1.07 (0.14)	1.02 (0.19)
**Alpha L**	1.35 (0.03)	1.38 (0.04)	1.37 (0.05)	1.36 (0.05)	1.36 (0.03)	1.37 (0.04)	1.35 (0.05)	1.37 (0.05)	1.35 (0.04)	1.34 (0.05)	1.31 (0.07)	1.32 (0.04)
**SD1 mm Hg**	1.86 (0.61)	2.75 (1.13)	2.11 (0.55)	2.56 (0.77)	2.06 (0.58)	2.83 (0.71)	2.47 (0.73)	3.09 (1.18)	2.25 (0.35)	3.25 (0.95)	2.45 (0.72)	4.3 (1.83)
**SD2 mm Hg**	21.7 (4.6)	22.4 (4.9)	24.8 (3.6)	21.6 (5.8)	25.7 (5.9)	29.2 (8.1)	26.6 (6.3)	26.9 (7.6)	23.3 (3.9)	21.4 (3.7)	24.1 (4.5)	25.6 (6.5)

Values are means (standard deviation); n = 9 control rats; n = 10 ouabain treated rats. Non-linear measures were determined in 35 min long segments. Data are averages of results of variability analysis in first two complete segments after 12 p.m. and 12 a.m. Alpha S, short-term fractal scaling exponent; Alpha L, long-term fractal scaling exponent; SD1, standard deviation of the Poincare plot in the direction of the normal of the diagonal (fast component); SD2, standard deviation of the Poincare plot in the direction of the diagonal (slow component).
(Statistical results are on next page).

Table S6. Complexity and self-similarity of heart rate variability (MANOVA results)

	Interactions								Main effects					
	3-way		Time x Group		Illumination x Group		Time x Illumination		Group		Time		Illumination	
	$F_{2,16}$	P	$F_{2,16}$	P	$F_{1,17}$	P	$F_{2,16}$	P	$F_{1,17}$	P	$F_{2,16}$	P	$F_{1,17}$	P
**Lempel-Ziv entropy**	0.6	0.565	4.7	0.025	2.4	0.140	2.8	0.881	4.6	0.048	0.3	0.759	67.5	10^{-6}
**Shannon's entropy**	0.9	0.425	7.9	0.005	4.6	0.049	6.8	0.009	0.1	0.716	0.5	0.647	9.9	0.007
**Fractal dimension**	1.0	0.409	6.6	0.008	1.0	0.341	1.5	0.255	0.4	0.541	0.9	0.42	38.1	10^{-6}
**Alpha S**	1.5	0.259	0.1	0.929	0.4	0.552	0.2	0.831	0.1	0.724	1.0	0.379	10.1	0.004
**Alpha L**	0.1	0.960	6.3	0.010	0.001	0.992	2.2	0.144	2.3	0.116	3.4	0.060	0.8	0.396
**SD1 mm Hg**	2.7	0.096	2.2	0.149	2.2	0.152	3.5	0.056	3.2	0.093	5.0	0.021	38.4	1.10^{-5}
**SD2 mm Hg**	0.5	0.647	6.3	0.01	0.1	0.833	4.5	0.027	0.1	0.838	3.6	0.052	0.05	0.825

Within groups main effects and their interactions were tested with repeated measures MANOVA and multivariate Wilks test; between groups main effect “group” was tested with the univariate ANOVA (between-within design; 2 levels of main effect “group” x 2 levels of main effect “illumination” x 3 levels of main effect “time/ouabain treatment”). Alpha S, short-term fractal scaling exponent; Alpha L, long-term fractal scaling exponent; SD1, standard deviation of the Poincare plot in the direction of the normal of the diagonal (fast component); SD2, standard deviation of the Poincare plot in the direction of the diagonal (slow component). F, multivariate (repeated measures factors) or univariate (between groups factor) F-test values, subscripts are degrees of freedom; P, probability
