# Supplementary material for: Wistar Rats Resistant to the Hypertensive Effects of Ouabain Exhibit Enhanced Cardiac Vagal Activity and Elevated Plasma Levels of Calcitonin Gene-Related Peptide
Source: PLoS One. 2014 Oct 3;9(10):e108909. doi: 10.1371/journal.pone.0108909 (PMC4184851; doi:10.1371/journal.pone.0108909)
Supplement: Table S7 — Baroreflex sensitivity. (PDF) [file pone.0108909.s012.pdf]

Table S7. Baroreflex sensitivity

	Control Ouabain 0 µg/kg Day 0		Day 20		Day 60		Ouabain treated 0 µg/kg Day 0		63 µg/kg Day 20		324 µg/kg Day 60	
	Dark	Light	Dark	Light	Dark	Light	Dark	Light	Dark	Light	Dark	Light
**Tachycardic sequences ms/mm Hg**	1.5 (0.3)	1.8 (0.6)	1.4 (0.3)	1.8 (0.4)	1.5 (0.2)	1.8 (0.5)	1.5 (0.3)	1.7 (0.3)	1.5 (0.2)	1.8 (0.3)	1.6 (0.3)	1.9 (0.3)
**Bradycardic sequences ms/mm Hg**	1.3 (0.2)	1.8 (0.6)	1.3 (0.3)	1.7 (0.4)	1.4 (0.2)	1.7 (0.4)	1.5 (0.4)	1.5 (0.3)	1.4 (0.3)	1.7 (0.3)	1.4 (0.4)	1.7 (0.3)
**Complex demodulation (ms/mm Hg)**	1.1 (0.3)	1.5 (0.7)	1.2 (0.3)	1.4 (0.5)	1.1 (0.3)	1.5 (0.4)	1.3 (0.4)	1.4 (0.4)	1.2 (0.3)	1.5 (0.4)	1.2 (0.2)	1.6 (0.4)
**Alpha coefficient LF (ms/mm Hg)**	2.0 (0.5)	2.5 (0.7)	1.9 (0.4)	2.5 (0.7)	2.1 (0.9)	2.1 (0.5)	2.2 (0.7)	2.4 (0.6)	1.8 (0.3)	2.6 (0.7)	2.2 (0.5)	2.7 (0.8)
**Alpha coefficient HF (ms/mm Hg)**	1.1 (0.2)	1.6 (0.6)	1.1 (0.3)	1.5 (0.4)	1.1 (0.2)	1.7 (0.5)	1.4 (0.4)	1.7 (0.5)	1.3 (0.3)	1.9 (0.5)	1.4 (0.3)	2.0 (0.4)

Values are means (standard deviation); n = 9 control rats; n = 10 ouabain treated rats. Baroreflex sensitivity measures were determined in 35 min long segments. Data are averages of results of variability analysis in first two complete segments after 12 p.m. and 12 a.m. Tachycardic sequences, minimum of 3 beats with declining systolic pressure associated with shortened RR-intervals; Bradycardic, sequences, minimum of 3 beats with increasing systolic pressure associated with prolonged RR-intervals; Complex demodulation, mean value of the ratio of the smoothed amplitude oscillations in RR-interval and systolic pressure; Alpha-index, square root of the ratio between RR-interval spectral powers and the systolic blood pressure spectral powers separately in the low (LF) and high frequency (HF) ranges.

(Statistical results are on next page)

Table S7. Baroreflex sensitivity (MANOVA results)

	Interactions								Main effects					
	3-way		Time x Group		Illumination x Group		Time x Illumination		Group		Time		Illumination	
	$F_{2,16}$	P	$F_{2,16}$	P	$F_{1,17}$	P	$F_{2,16}$	P	$F_{1,17}$	P	$F_{2,16}$	P	$F_{1,17}$	P
**Tachycardic sequences ms/mm Hg**	2.7	0.100	0.2	0.829	3.7	0.072	0.54	0.593	10^{-3}	0.974	0.5	0.621	20.8	3.10^{-4}
**Bradycardic sequences ms/mm Hg**	0.7	0.518	0.7	0.508	0.8	0.400	0.4	0.680	10^{-4}	0.992	1.4	0.271	36.1	10^{-5}
**Complex demodulation (ms/mm Hg)**	1.3	0.295	0.5	0.597	47.9	2.10^{-6}	1.2	0.341	0.1	0.717	0.6	0.570	47.9	2.10^{-6}
**Alpha coefficient LF (ms/mm Hg)**	1.4	0.275	0.4	0.696	27.7	6.10^{-5}	3.5	0.053	0.4	0.514	0.1	0.941	27.7	6.10^{-5}
**Alpha coefficient HF (ms/mm Hg)**	1.2	0.317	0.5	0.644	0.02	0.879	1.3	0.261	4.1	0.058	2.3	0.132	75.0	10^{-6}

Within groups main effects and their interactions were tested with repeated measures MANOVA and multivariate Wilks test; between groups main effect "group" was tested with the univariate ANOVA (between-within design; 2 levels of main effect "group" x 2 levels of main effect "illumination" x 3 levels of main effect "time/ouabain treatment"). Tachycardic sequences, minimum of 3 beats with declining systolic pressure associated with shortened RR-intervals; Bradycardic, sequences, minimum of 3 beats with increasing systolic pressure associated with prolonged RR-intervals; Complex demodulation, mean value of the ratio of the smoothed amplitude oscillations in RR-interval and systolic pressure; Alpha-index, square root of the ratio between RR-interval spectral powers and the systolic blood pressure spectral powers separately in the low (LF) and high frequency (HF) ranges. F, multivariate (repeated measures factors) or univariate (between groups factor) F-test values, subscripts are degrees of freedom; P, probability.
