# Supplementary material for: Wistar Rats Resistant to the Hypertensive Effects of Ouabain Exhibit Enhanced Cardiac Vagal Activity and Elevated Plasma Levels of Calcitonin Gene-Related Peptide
Source: PLoS One. 2014 Oct 3;9(10):e108909. doi: 10.1371/journal.pone.0108909 (PMC4184851; doi:10.1371/journal.pone.0108909)
Supplement: Table S8 — Time- and frequency-domain indices of systolic pressure variability. (PDF) [file pone.0108909.s013.pdf]

Table S8. Time and frequency domain indices of systolic pressure variability

	Control						Ouabain treated					
	Ouabain 0 µg/kg						0 µg/kg		63 µg/kg		324 µg/kg	
	Day 0		Day 20		Day 60		Day 0		Day 20		Day 60	
	Dark	Light	Dark	Light	Dark	Light	Dark	Light	Dark	Light	Dark	Light
**SP mmHg**	137 (9)	132 (8)	137 (9)	130 (6)	140 (10)	136 (8)	139 (7)	134 (6)	136 (10)	129 (7)	137 (10)	130 (10)
**SD of SPmmHg**	7 (1)	6 (1)	8 (1)	6 (1)	8 (2)	8 (1)	8 (1)	7 (1)	7 (1)	5 (1)	8 (1)	7 (1)
**TP lnAUC $mmHg^2.s$**	10.5 (0.2)	10.2 (0.2)	10.5 (0.1)	10.1 (0.2)	10.6 (0.3)	10.4 (0.3)	10.5 (0.1)	10.3 (0.2)	10.4 (0.2)	10.0 (0.2)	10.5 (0.2)	10.3 (0.3)
**VLF lnAUC $mmHg^2.s$**	10.1 (0.3)	9.9 (0.2)	10.2 (0.1)	9.9 (0.2)	10.1 (0.3)	10.2 (0.4)	10.1 (0.2)	10.0 (0.2)	10.0 (0.2)	9.6 (0.2)	10.1 (0.3)	9.9 (0.3)
**LF lnAUC $mmHg^2.s$**	9.1 (0.2)	8.5 (0.2)	9.0 (0.2)	8.4 (0.3)	9.0 (0.2)	8.7 (0.3)	9.2 (0.2)	8.7 (0.3)	9.2 (0.2)	8.5 (0.3)	9.0 (0.2)	8.8 (0.2)
**HF lnAUC $mm\ Hg^2.s$**	7.3 (0.1)	6.9 (0.2)	7.3 (0.1)	6.9 (0.3)	7.3 (0.2)	7.0 (0.3)	7.2 (0.2)	7.0 (0.1)	7.2 (0.3)	6.8 (0.3)	7.3 (0.3)	7.0 (0.4)

Values are means (standard deviation); n = 9 control rats; n = 10 ouabain treated rats. SP, systolic pressure; SD, standard deviation of SP; ln, natural logarithm; AUC, area under curve; TP, total power of systolic pressure variability; VLF, very low frequency power of systolic pressure variability; LF, low frequency power of systolic pressure variability; HF, high frequency power of systolic pressure variability. Spectral powers were determined in 2s intervals (Wigner-Ville transform) in 35 min long segments, then integrated over the whole 35 min and logarithmically transformed. Data are averages of results of variability analysis in first two complete segments after 12 p.m. and 12 a.m.

(Statistical results are on next page).

Table S8. Time and frequency domain indices of systolic pressure variability (MANOVA results)

	Interactions								Main effects					
	3-way		Time x Group		Illumination x Group		Time x Illumination		Group		Time		Illumination	
	$F_{2,16}$	P	$F_{2,16}$	P	$F_{1,17}$	P	$F_{2,16}$	P	$F_{1,17}$	P	$F_{2,16}$	P	$F_{1,17}$	P
**SP mmHg**	1.4	0.266	3.5	0.056	0.6	0.471	1.3	0.284	0.1	0.82	11.6	8.10^{-4}	51.5	2.10^{-6}
**SD of SPmmHg**	1.5	0.256	9.5	2.10^{-3}	0.1	0.709	6.1	0.011	0.5	0.506	6.5	8.10^{-3}	49.2	2.10^{-6}
**TP lnAUC mmHg^2.s**	1.9	0.177	4.0	0.04	0.126	0.727	7.3	0.006	0.2	0.683	9.9	0.002	57.2	10^{-6}
**VLF lnAUC mmHg^2.s**	1.9	0.176	6.6	0.008	0.8	0.374	5.9	0.012	1.9	0.179	7.9	0.004	33.3	2.10^{-5}
**LF lnAUC mmHg^2.s**	0.5	0.622	0.9	0.446	0.03	0.873	10.7	0.001	2.2	0.156	5.4	0.015	140.1	10^{-7}
**HF lnAUC mm Hg^2.s**	1.9	0.185	0.3	0.768	0.4	0.538	5.7	0.014	0.03	0.862	2.9	0.084	50.5	2.10^{-6}

Within groups main effects and their interactions were tested with repeated measures MANOVA and multivariate Wilks test; between groups main effect “group” was tested with the univariate ANOVA (between-within design; 2 levels of main effect “group” x 2 levels of main effect “illumination” x 3 levels of main effect “time/ouabain treatment”). SP, systolic pressure; SD, standard deviation; ln, natural logarithm; AUC, area under curve; TP, total power of systolic pressure variability; VLF, very low frequency power of systolic pressure variability; LF, low frequency power of systolic pressure variability; HF, high frequency power of systolic pressure variability; F, multivariate (repeated measures factors) or univariate (between groups factor) F-test values, subscripts are degrees of freedom; P, probability.
