# Supplementary material for: Wistar Rats Resistant to the Hypertensive Effects of Ouabain Exhibit Enhanced Cardiac Vagal Activity and Elevated Plasma Levels of Calcitonin Gene-Related Peptide
Source: PLoS One. 2014 Oct 3;9(10):e108909. doi: 10.1371/journal.pone.0108909 (PMC4184851; doi:10.1371/journal.pone.0108909)
Supplement: Table S9 — Complexity and self-similarity of systolic pressure variability. (PDF) [file pone.0108909.s014.pdf]

Table S9. Complexity and self-similarity of systolic pressure variability

	Control						Ouabain treated					
	Ouabain 0 µg/kg						0 µg/kg		63 µg/kg		324 µg/kg	
	Day 0		Day 20		Day 60		Day 0		Day 20		Day 60	
	Dark	Light	Dark	Light	Dark	Light	Dark	Light	Dark	Light	Dark	Light
**Lempel-Ziv entropy**	0.44 (0.06)	0.49 (0.03)	0.41 (0.05)	0.49 (0.05)	0.42 (0.1)	0.45 (0.04)	0.42 (0.05)	0.49 (0.09)	0.44 (0.05)	0.55 (0.08)	0.42 (0.1)	0.52 (0.09)
**Shannon's entropy**	0.62 (0.03)	0.63 (0.03)	0.59 (0.03)	0.62 (0.04)	0.6 (0.04)	0.58 (0.04)	0.58 (0.06)	0.62 (0.07)	0.59 (0.06)	0.66 (0.08)	0.55 (0.08)	0.63 (0.08)
**Fractal dimension**	1.7 (0.04)	1.72 (0.03)	1.69 (0.03)	1.72 (0.04)	1.68 (0.05)	1.7 (0.05)	1.72 (0.02)	1.75 (0.03)	1.72 (0.03)	1.78 (0.03)	1.72 (0.03)	1.76 (0.04)
**Alpha S**	0.94 (0.05)	1.02 (0.05)	0.96 (0.06)	1.03 (0.07)	0.97 (0.04)	1.02 (0.07)	0.9 (0.04)	0.95 (0.05)	0.91 (0.05)	0.93 (0.04)	0.92 (0.06)	0.97 (0.08)
**Alpha L**	1.35 (0.07)	1.25 (0.08)	1.32 (0.09)	1.25 (0.11)	1.32 (0.1)	1.3 (0.14)	1.42 (0.08)	1.3 (0.13)	1.39 (0.08)	1.29 (0.15)	1.37 (0.07)	1.25 (0.16)
**SD1 mmHg**	1.75 (0.11)	1.63 (0.15)	1.71 (0.13)	1.57 (0.21)	1.68 (0.12)	1.63 (0.22)	1.71 (0.12)	1.84 (0.26)	1.71 (0.19)	1.71 (0.33)	1.77 (0.26)	1.92 (0.5)
**SD2 mmHg**	10.16 (1.57)	8.44 (0.91)	11.47 (1.35)	8.22 (0.8)	11.32 (2.53)	10.67 (1.78)	10.68 (0.99)	9.4 (1.48)	10.48 (1.87)	7.38 (0.63)	11.18 (1.65)	9.12 (1.68)

Values are means (standard deviation); n = 9 control rats; n = 10 ouabain treated rats. Non-linear measures were determined in 35 min long segments. Data are averages of results of variability analysis in first two complete segments after 12 p.m. and 12 a.m. Alpha S, short-term fractal scaling exponent; Alpha L, long-term fractal scaling exponent; SD1, standard deviation of the Poincare plot in the direction of the normal of the diagonal (fast component); SD2, standard deviation of the Poincare plot in the direction of the diagonal (slow component).

(Statistical results are on next page)

Table S9. Complexity and self-similarity of systolic pressure variability (MANOVA results)

	Interactions								Main effects					
	3-way		Time x Group		Illumination x Group		Time x Illumination		Group		Time		Illumination	
	$F_{2,16}$	P	$F_{2,16}$	P	$F_{1,17}$	P	$F_{2,16}$	P	$F_{1,17}$	P	$F_{2,16}$	P	$F_{1,17}$	P
**Lempel-Ziv entropy**	1.4	0.276	2.6	0.108	1.9	0.182	1.7	0.222	1.7	0.215	1.1	0.348	27.4	7.10^{-5}
**Shannon's entropy**	2.1	0.153	2.8	0.091	5.5	0.031	1.3	0.311	0.01	0.621	1.6	0.242	8.5	0.01
**Fractal dimension**	1.1	0.361	4.1	0.367	4.4	0.051	3.4	0.157	1.3	0.277	1.8	0.189	36.9	10^{-5}
**Alpha S**	0.6	0.541	2.5	0.149	1.3	0.277	1.21	0.323	1.9	0.211	2.3	0.134	30.1	4.10^{-5}
**Alpha L**	1.1	0.354	3.3	0.062	1.7	0.212	1.6	0.239	0.9	0.348	1.3	0.296	24.9	10^{-4}
**SD1 mmHg**	0.9	0.411	1.2	0.318	5.1	0.036	2.9	0.086	1.5	0.231	2.9	0.083	0.001	0.988
**SD2 mmHg**	1.5	0.254	9.7	0.002	0.2	0.631	6.1	0.011	0.6	0.464	6.4	0.009	51.5	2.10^{-6}

Within groups main effects and their interactions were tested with repeated measures MANOVA and multivariate Wilks test; between groups main effect “group” was tested with the univariate ANOVA (between-within design; 2 levels of main effect “group” x 2 levels of main effect “illumination” x 3 levels of main effect “time/ouabain treatment”). Alpha S, short-term fractal scaling exponent; Alpha L, long-term fractal scaling exponent; SD1, standard deviation of the Poincare plot in the direction of the normal of the diagonal (fast component); SD2, standard deviation of the Poincare plot in the direction of the diagonal (slow component). F, multivariate (repeated measures factors) or univariate (between groups factor) F-test values, subscripts are degrees of freedom; P, probability.
