# Supplementary material for: Wistar Rats Resistant to the Hypertensive Effects of Ouabain Exhibit Enhanced Cardiac Vagal Activity and Elevated Plasma Levels of Calcitonin Gene-Related Peptide
Source: PLoS One. 2014 Oct 3;9(10):e108909. doi: 10.1371/journal.pone.0108909 (PMC4184851; doi:10.1371/journal.pone.0108909)
Supplement: Table S10 — Hemodynamic responses to restraint stress (MANOVA results). (PDF) [file pone.0108909.s015.pdf]

Table S10. Hemodynamic responses to restraint stress (MANOVA results)

	Interactions								Main effects					
	3-way		Time x Group		Salt intake x Group		Time x Salt intake		Group		Time		Salt intake	
	$F_{2,16}$	P	$F_{2,16}$	P	$F_{1,17}$	P	$F_{2,16}$	P	$F_{1,17}$	P	$F_{2,16}$	P	$F_{1,17}$	P
**Δ $MAP_{maximal}$ mmHg**	0.21	0.813	1.2	0.337	0.30	0.618	1.4	0.282	1.53	0.233	2.6	0.107	1.9	0.189
**Δ $MAP_{restraint}$ mmHg**	0.3	0.737	0.9	0.416	0.10	0.830	0.9	0.427	2.1	0.166	0.7	0.520	2.3	0.144
**Δ $MAP_{recovery}$ mmHg**	0.7	0.508	4.0	0.040	0.13	0.727	7.3	0.006	0.1	0.817	1.0	0.390	2.8	0.130
**Δ $HR_{maximal}$ beats/min**	0.9	0.430	5.2	0.019	0.03	0.865	0.7	0.494	0.1	0.770	2.5	0.112	3.7	0.070
**Δ $HR_{restraint}$ beats/min**	0.4	0.650	3.1	0.079	0.01	0.839	1.0	0.378	0.8	0.387	2.7	0.1	2.9	0.107
**Δ $HR_{recovery}$ beats/min**	1.7	0.206	1.3	0.294	1.3	0.271	0.3	0.744	0.5	0.474	1.1	0.352	1.5	0.241

Within groups main effects and their interactions were tested with repeated measure MANOVA and multivariate Wilks test; between groups main effect "group" was tested with the univariate ANOVA (between-within design with 2 levels of the main effect "group" x 3 levels of the main effect "time/ouabain treatment" x 2 levels of the main effect "salt intake"). MAP, mean arterial pressure; HR, heart rate; Δ maximal, difference between the maximal value during the restraint and averaged values during the period of 10 min before the restraint; Δ restraint, difference between the averaged values during the first 10 min of restraint and averaged values during the period of 10 min before the restraint; Δ recovery, difference between the averaged values during the 21st - 30th min of recovery and averaged values during the period of 10 min before the restraint; F, multivariate (repeated measures factors) or univariate (between groups factor) F-test values, subscripts are degrees of freedom; P, probability.
