# Supplementary material for: Wistar Rats Resistant to the Hypertensive Effects of Ouabain Exhibit Enhanced Cardiac Vagal Activity and Elevated Plasma Levels of Calcitonin Gene-Related Peptide
Source: PLoS One. 2014 Oct 3;9(10):e108909. doi: 10.1371/journal.pone.0108909 (PMC4184851; doi:10.1371/journal.pone.0108909)
Supplement: Table S11 — Blood pressure and heart rate variability responses to restraint stress (MANOVA results). (PDF) [file pone.0108909.s016.pdf]

## Table S11. Blood pressure and heart rate variability responses to restraint stress (MANOVA results)

| | Interactions | | | | | | | | Main effects | | | | | |
|---|---|---|---|---|---|---|---|---|---|---|---|---|---|---|
| | 3-way | | Time x Group | | Salt intake x Group | | Time x Salt intake | | Group | | Time | | Salt intake | |
| | $F_{2,16}$ | P | $F_{2,16}$ | P | $F_{1,17}$ | P | $F_{2,16}$ | P | $F_{1,17}$ | P | $F_{2,16}$ | P | $F_{1,17}$ | P |
| **LF $SP_{baseline}$ mmHg** | 1.6 | 0.223 | 2.4 | 0.120 | 0.5 | 0.511 | 2.1 | 0.160 | 0.9 | 0.358 | 0.1 | 0.902 | 29.1 | $5.10^{-5}$ |
| **Δ LF $SP_{restraint}$ mmHg** | 0.2 | 0.792 | 1.7 | 0.208 | 0.2 | 0.700 | 0.1 | 0.918 | 1.9 | 0.183 | 3.1 | 0.075 | 4.2 | 0.057 |
| **Δ LF $SP_{recovery}$ mmHg** | 2.4 | 0.124 | 1.3 | 0.301 | 0.1 | 0.818 | 0.4 | 0.700 | 0.2 | 0.625 | 0.1 | 0.920 | 0.3 | 0.602 |
| **HF $RRI_{baseline}$ beats/min** | 0.8 | 0.449 | 0.7 | 0.492 | 0.9 | 0.362 | 0.3 | 0.742 | 0.8 | 0.386 | 0.1 | 0.887 | 0.5 | 0.476 |
| **Δ HF $RRI_{restraint}$ beats/min** | 1.1 | 0.351 | 1.7 | 0.210 | 2.1 | 0.169 | 0.1 | 0.958 | 0.1 | 0.848 | 1.5 | 0.265 | 1.1 | 0.319 |
| **Δ HF $RRI_{recovery}$ beats/min** | 2.7 | 0.101 | 0.2 | 0.797 | 0.1 | 0.776 | 1.7 | 0.223 | 0.6 | 0.437 | 0.1 | 0.867 | 0.6 | 0.465 |

Within groups main effects and their interactions were tested with repeated measure MANOVA and multivariate Wilks test; between groups main effect "group" was tested with the univariate ANOVA (between-within design with 2 levels of the main effect "group" x 3 levels of the main effect "time/ouabain treatment" x 2 levels of the main effect "salt intake"; LF SP, low frequency power of systolic pressure variability; HF RRI, high frequency power of RR-interval variability; baseline, averaged variability during the period of 10 min before the restraint; Δ restraint, difference between the averaged values during the first 10 min of restraint and averaged values during the period of 10 min before the restraint; Δ recovery, difference between the averaged values during the 21st - 30th min of recovery and averaged values during the period of 10 min before the restraint; F, multivariate (repeated measures factors) or univariate (between groups factor) F-test values, subscripts are degrees of freedom; P, probability.
