# Supplementary material for: Wistar Rats Resistant to the Hypertensive Effects of Ouabain Exhibit Enhanced Cardiac Vagal Activity and Elevated Plasma Levels of Calcitonin Gene-Related Peptide
Source: PLoS One. 2014 Oct 3;9(10):e108909. doi: 10.1371/journal.pone.0108909 (PMC4184851; doi:10.1371/journal.pone.0108909)
Supplement: Methods S1 — Detailed description of the experimental procedures. (PDF) [file pone.0108909.s017.pdf]

Detailed description of methods

Experimental protocol

Animals were implanted with telemetric transmitters and were allowed a minimum of 3 weeks of recovery before starting the experimental protocol. This recovery period permitted for full normalization of heart rate, blood pressure and body temperature circadian rhythms.

Baseline data were collected as three full day recordings (days -8, -4, 0) before administration of the first dose of ouabain on day 0. Thereafter data were collected each fourth day for 20 days followed by a "testing period" of 20 days. Telemetry monitoring interval of four days was selected to ensure that batteries could provide power over the whole 3 months long experimental period. Multiple reports indicating at least 2-weeks delay between start of ouabain administration and hypertension appearance (Table S1). This makes improbable that ouabain-induced hypertension could be missed because of too infrequent monitoring.

On day 40, a second dose of ouabain was applied; data were collected each fourth day for 20 additional days and the testing period was repeated. At the end of the experiment, rats were sacrificed by exsanguination under carbon dioxide anesthesia and tissues and plasma were collected for subsequent gene expression and ELISA analysis. The testing protocol was applied three times; once before taking the baseline data and then twice after completion of twenty days administration of each ouabain dose. During the testing period, the response to ganglionic blockade and α-1 adrenergic stimulation was examined, followed by salt sensitivity and restraint tests and tail-cuff blood pressure measurement simulation.

Experimental animals

Rats were kept individually in transparent polycarbonate cages (42 x 29 x 19 cm) in an environmentally controlled room (22 ± 1°C; 12 h light-dark cycles, dark period started at 7:00 a.m.). Bedding (Bed-o'Cobs, #8B, Andersons lab bedding, Maumee, OH) was changed twice a week. Animals had free access to food (0.63% NaCl, 801700, SDS, Essex, UK) and water and their welfare was assessed twice a week by taking body weights, measuring food and water consumption and observing their behavior. Required sample size was estimated based on power analysis given alpha error probability 0.05, power 0.8 and effect size 0.8. Calculations were done with the G*Power 3.1.2 software [1].

Administration of ouabain

Ouabain was administered in two doses of 63 (3, S.D.) and 324 (21, S.D.) μg of ouabain /kg/day by subcutaneous implantation of 1.5 and 7.5 mg 90-day release ouabain pellets (SC-331, Innovative research of America, Sarasota, FL). The average ouabain dose was calculated taking into account changes in body weight during the experiment.

Implantation of transmitters

Before implantation, transmitters were switched on for 24 hours and the precision of zero pressure assessment was checked. To correct for possible drift, the zero pressure offset was checked again after explantation. All recorded pressure values were corrected for the determined drift before any further processing. Recorded drift was between -0.8 to -1.3 mm Hg / month.

Rats were anesthetized with *i.p.* injection of a mixture of ketamine hydrochloride (120 mg/kg, HIKMA Pharmaceuticals, Jordan), xylazine hydrochloride (6 mg/kg, Sigma, USA) and atropine

sulphate monohydrate (0.12 mg/kg, Sigma, USA). The body of the transmitter (TL11M2-C50-PXT, Data Sciences, St. Paul, MN, USA) was placed intraperitoneally, pressure catheter was introduced into the abdominal aorta above the iliac bifurcation and caudal to the renal arteries via the femoral artery, and ECG leads were placed into the upper mediastinum and on the left thorax side underneath the pectoralis minor.

Arterial blood pressure and heart rate variability

Arterial blood pressure and heart rate variability were analyzed in 35-min-long segments with WinCPRS software (Absolute Aliens, Turku, Finland). First two complete segments after 12 a.m. were analyzed for the light period and first two complete segments after 12 p.m. were analyzed for the dark period. Systolic pressure and RR-interval time series were manually checked for recording, movement, or identification artifacts as well as for arrhythmias before further processing. Disturbances in the sinus rhythm were corrected by interpolation, i.e. previous and next correct RRI values were used to adjust the abnormal beats. Stationarity testing showed that both systolic pressure and RR-interval time series had unacceptable levels of non-stationarity. Therefore, instead of standard fast Fourier spectral analysis, time-frequency approach based on Wigner-Ville distribution was used to calculate power spectra of systolic pressure and RR-interval variability in short (2s) time intervals. Frequency ranges for the calculation of spectral powers were adjusted for rats heart rate, i.e. very low frequency band (0.02 to 0.2 Hz), low frequency band (0.2 to 0.7 Hz), high frequency band (0.7 to 3 Hz) and 0-3 Hz for the total power. Finally, all spectral powers within the 35-min-long segments were integrated as the area under the curve (AUC). Because the distribution of spectral powers was not normal, AUC-values were logarithmically transformed before statistical analysis.

Complexity and fractal properties of the RR-interval and systolic pressure time series were estimated by calculating Lempel-Ziv and Shannon's entropy [2], fractal scaling exponents [3], fractal dimension [2] and Poincare (return) plots [4].

Baroreflex sensitivity

To avoid interference of stress and vasoactive drugs with baroreflex sensitivity measurements, non-invasive estimation of spontaneous baroreflex sensitivity based on simultaneous analysis of spontaneous fluctuations in blood pressure and RR-intervals was performed. Although the information is probably not the same as that provided by the pharmacological method, it measures baroreflex sensitivity in the physiological blood pressure ranges over a period of time rather than during the brief and extreme blood pressure perturbations induced by pharmacological maneuvers. Three computationally different methods of spontaneous baroreflex assessment were used to minimize the risk of computational artifacts. All spontaneous baroreflex sensitivity measures were calculated with the WinCPRS software.

In the sequence method [5], ramps of decreasing or increasing systolic blood pressure values of minimally 3 beats were identified and slopes between the systolic blood pressure ramps and RR-intervals were determined. Baroreflex gain was calculated as the mean of all slopes where the systolic blood pressure changes were > 0.5 mm Hg, RR-interval changes > 1 ms and correlation coefficient > 0.85. Baroreflex sensitivity was calculated separately for the tachycardic sequences, i.e. sequences of blood pressure decreases followed by an increase in heart rate and bradycardic sequences, i.e. sequences of blood pressure increases followed by a decrease in heart rate. Baroreflex sensitivity by complex demodulation [6] was estimated as the mean value of the ratio of the smoothed amplitude oscillations in demodulated systolic blood pressure and RR-

intervals time series in the low frequency band (0.2-0.7 Hz). This method estimates baroreflex sensitivity continuously during very short (< 1 s) time intervals. Spectral analysis by the fast Fourier transform of systolic pressure and RR-interval time series was first performed to compute spontaneous baroreflex sensitivity by the alpha method [7]. Alpha coefficient in the low frequency band was defined as the square root of the ratio between systolic pressure and RR-interval spectra in the frequency range of 0-0.7 Hz but only over those frequencies where coherence between the systolic pressure and RR-interval time series was larger than 0.5. Similarly, alpha coefficient in the high frequency band was defined as the square root of the ratio between systolic pressure and RR-interval spectra in the frequency range of 0.7-3 Hz where the coherence condition was fulfilled.

Pharmacological estimation of arteriolar sympathetic activity

After 2 hours of baseline data collection, the ganglionic blocker hexamethonium bromide (30 mg/kg, Sigma-Aldrich, St. Louis, MO, USA) was injected subcutaneously to interrupt the vascular sympathetic drive and data were collected for 15 min. The magnitude of the difference between the blood pressure values before and after ganglionic blockade should reflect the contribution of sympathetic stimulation to arteriolar constriction. Subsequently, the alpha-1 adrenergic receptor agonist phenylephrine hydrochloride (3 mg/kg, Sigma-Aldrich) was subcutaneously administered and data were collected for another 2 hours. The aim of this maneuver was to look for postulated ouabain-induced changes in alpha-1 adrenergic receptors.

Estimation of salt sensitivity

Rats were transferred to metabolic cages that allow telemetric recording (CX-1300, BASI, Bioanalytical systems Inc. Indiana, USA) and were given distilled deionized water and sodium deficient diet (0.08% NaCl, TD.90228, Harlan Teklad, Wisconsin, USA) in the form of paste. Metabolic cages were cleaned twice a day (6:30-7:00 and 18:30-19:00) during which food and water consumption in addition to urinary output were measured. Rats were allowed a period of 5 days to adapt to the new conditions. On day 6, restraint stress test was performed followed by a full day recording of data on day 7. On day 8, the diet was changed to high salt diet (8% NaCl, TD.92012, Harlan Teklad). High salt diet was given only for 2 days to avoid potential renal damage [8]. Finally, restraint stress test was performed again but now with the rats being fed a high salt diet. After the completion of salt sensitivity and stress testing, rats were returned to their original cages with regular food and water. Urine was collected at the end of the dark and light periods respectively. A portable vial chiller (i-cup, CX-1400, BASI, Bioanalytical systems, Indiana, USA) was used to refrigerate urine as it was collected. The i-cup chiller (after being cooled at -70°C for 6 hours) is able to keep the collected urine cold below 4°C for a minimum of 16 hours. Once collected, urine volume was measured and aliquots were stored at -70°C for subsequent ELISA analysis.

Restraint stress test

Before the beginning of the experimental protocol, rats were trained to restraining stress. This was done by keeping the rats in restrainers for 30 min on alternate days; habituation was confirmed by stable cardiovascular response to restraint. Cylindrical, plexiglas, transparent restrainers designed for rats weighing 300-500 g were used. Restrainers were washed before each usage to prevent any stress effect mediated by odor remaining from previously restrained rats. During the restraint stress test, two hours of baseline recordings were performed. Thereafter, rats

were kept in restrainers for 30 min after which they were released and recording proceeded for 2 more hours of recovery.

Sample collection

Plasma. Rats were anesthetized with CO_2 and the abdomen was cut horizontally just below the diaphragm. The ribcage was cut vertically parallel to the sternum to expose the heart. Blood was withdrawn from the left ventricular cavity into heparinized vacutainers (#BD367876, GP supplies, London, UK). Blood was cold centrifuged (4°C) at 1207xg for 10 min after which aliquots of plasma were stored at -70°C until processed.

Tissues. Immediately after blood withdrawal, the heart and aorta were excised together and washed with sterile ice-cold saline. Then they were kept on ice and cleaned from surrounding fat and connective tissue. Aorta was separated and heart was weighed and dissected into atria, right ventricle, left ventricle and septum. During this time, the superior mesenteric artery with the first order arcade vessels was isolated and cleaned. Furthermore, the left kidney was dissected out and separated into cortex and medulla. All tissues were kept in sterile cryotubes (#89020, TPP Technoplastic products, Trasadingen, Switzerland), frozen in liquid nitrogen and stored at -70°C.

Urine. was collected twice a day (dark and light samples) during the 3 days of salt sensitivity test when the rats were housed in metabolic cages. A portable, non-electric vial chiller (i-cup, CX-1400, BASI, Bioanalytical systems Inc., Indiana, USA) was used to refrigerate urine as it was collected. The i-cup chiller, when kept at -70°C for 6 hours, is able to keep the collected urine cold below 4°C for a minimum of 16 hours. Once collected, urine volume was measured and aliquots were kept in eppendorf tubes and stored at -70°C for subsequent ELISA analysis. During high salt intake when urinary flow rate was high, urine was collected several times during the dark period.

RNA extraction and reverse transcription

Total RNA extraction was performed with TRIzol Reagent according to the manufacturer's instructions (15596-018, Invitrogen, NY). RNA concentration was measured using a Micro-Volume UV-Vis Spectro-photometer (NanoDrop 2000, Thermo Fisher Scientific Inc., Delaware). DEPC-water was used as a blank. A volume of 0.5 µl of each sample was used in duplicates and the absorbance was measured at 260 and 280 nm. The ratio A_{260}/A_{280} of ≥ 1.7 was indicative of the purity of the preparation. RNA concentration (µg/ml) was determined automatically by the software using the absorbance read at A_{260}. RNA integrity was assessed by running an aliquot (2 µg) of the RNA sample on a denaturing agarose gel stained with ethidium bromide [modified from 9]. 5X MOPS buffer (20.6 g MOPS, 3.28 g Na acetate, 10 ml of 0.5 M EDTA and DEPC water up to 1 liter) and 1% w/v agarose gel (0.5 g agarose, 10 ml 5X MOPS buffer, 30.5 ml DEPC water and 9 ml formaldehyde) were used. The samples were stained by adding 2 µl of sample to 8 µl sample mix (33 µl 5X MOPS buffer, 58 µl formaldehyde, 166.5 µl formamide and 0.5 µl ethidium bromide). The gel was run at 100V at room temperature using Bio-rad PowerPac 3000 (Bio-rad laboratories inc., USA). The resulting 28S rRNA and 18S rRNA bands were visualized under UV light using GeneGenius BioImaging System (Syngene, UK). RNA was considered intact if the bands were sharp, clear and the intensity ratio between 28S rRNA and 18S rRNA was 2:1. Thereafter, DNase treatment was performed followed by reverse transcription as described in [10].

Quantitative polymerase chain reaction (qPCR)

The qPCR reaction was carried out in a real-time PCR thermal cycler (model 7500, Applied Biosystems, Grand Island, NY). Probe-based qPCR (Taqman hydrolysis probes) was used. The search engine “Genevestigator” (www.refgenes.org) was used to select potential reference genes (RGs). Beta-actin (4352340E), 18S (4319413E) and GAPDH (4308313) were tentatively selected and purchased from Applied Biosystems and the stability of their expression in control and ouabain-treated animals verified by comparing Cq values separately for each gene. No significant differences in expression were detected for any of these genes. Because beta-actin had the smallest non-significant F-statistic, it was selected as a RG for subsequent qPCR analysis. The PCR reactions were prepared using the TAQMAN gene expression master mix (4369016, Applied Biosystems). Study samples were run in duplicate in MicroAmp optical 96-well reaction plates (4306737, Applied Biosystems). On ice, the following were added: 12.5 µl of 2x TAQMAN, 1.25 µl of GOI probe, 1.25 µl of RG probe, 1 µl of template cDNA (50 ng), 9 µl of sterile water. The PCR was then run as follows: 1 cycle of 2 min at 50°C; 1 cycle of 10 min at 95°C, followed by 40 cycles of 15 s at 95°C and 1 min at 60°C. In all experiments, 2 negative controls were run parallel to the samples; an RT- (where reverse transcriptase was not added) and a no template control (NTC, where water was added instead of sample), the former to exclude genomic DNA contamination and the latter to exclude reagent contamination and any amplification due to primer-dimer formation. The relative gene expression analysis with the comparative Cq (ΔΔCq) method adjusted for amplification efficiency was used to compare expression of GOI to a RG in control and ouabain-treated rats [11].

Amplification efficiencies (E) and Cq were derived from the raw fluorescence data with LinReg software [12, 13]. This program determines the baseline fluorescence per sample by reconstructing the log-linear phase of amplification. After the baseline correction, linear regression analysis of a subset of data points in the exponential phase is performed to determine the amplification efficiency per sample. Then the fluorescence threshold is placed in the upper part of the log-linear phase and the Cq value is determined.

Specifications of primers

Target	Assay ID	Gene symbol	Gene name	Reference sequence	NCBI Location Chromosome	Amplicon length
Na^+/Ca^{2+}-exchanger-1	Rn01472845_m1	Slc8a1	solute carrier family 8 (sodium/calcium exchanger), member1	NA	Chr.6: 4421046 - 4691716	69
α1 Na^+/K^+-ATPase	Rn01533986_m1	Atp1a1	ATPase, Na+/K+ transporting, alpha 1 polypeptide	NM_012504.1	Chr.2: 196657749 - 196687242	72
α2 Na^+/K^+-ATPase	Rn00560789_m1	Atp1a2	ATPase, Na+/K+ transporting, alpha 2 polypeptide	NM_012505.1	Chr.13: 88258993 - 88283918	68
α3 Na^+/K^+-ATPase	Rn00560813_m1	Atp1a3	ATPase, Na+/K+ transporting, alpha 3 polypeptide	NM_012506.1	Chr.1: 80280714 - 80309834	63
Cyclooxygenase1, COX1, constitutive	Rn00566881_m1	Ptgs1	prostaglandin-endoperoxide synthase 1	NM_017043.3	Chr.3: 15343832 - 15365412	85
Cyclooxygenase2, COX2, inducible	Rn01483828_m1	Ptgs2	prostaglandin-endoperoxide synthase 2	NM_017232.3	Chr.13: 64427288 - 64432978	112
Nitric oxide synthase1, NOS1	Rn00583793_m1	Nos1	nitric oxide synthase 1, neuronal	NM_052799.1	Chr.12: 39812500 - 39869484	65
Nitric oxide synthase2, NOS2	Rn00561646_m1	Nos2	nitric oxide synthase 2, inducible	NM_012611.3	Chr.10: 65036884 - 65072453	77
Nitric oxide synthase3, NOS3	Rn02132634_s1	Nos3	nitric oxide synthase 3, endothelial cell	NM_021838.2	Chr.4: 6158847 - 6179441	117

Liquid chromatography- tandem mass spectrometry analysis of ouabain and digoxin

Liquid chromatography-tandem mass spectrometry (LC/MS) using Quattro LC equipment (Micromass, Manchester, UK) in MS scanning mode was used. Samples were directly infused to the ionization probe using negative electrospray ionization (-ESI) in the mass range m/z 100-800 under optimized tuning conditions. For ouabain the limit of quantitation was 3.4 nmol /ml.

Reference stock solution of 6 mg/ml ouabain was prepared in methanol. A 100 µl aliquot of solution was diluted to 1 ml with mobile phase (acetonitrile/1 mmol ammonium acetate solution 80/20) (1 µmol/ml). Reference stock solution of 5 mg/ml digoxin was prepared in methanol. A 100 µl of solution was diluted to 1 ml with mobile phase (0.64 µmol/ml). Sample preparation: Ouabain 10 mg pellet was powdered and extracted by sonication with 1 ml of methanol and filtered using Millipore 0.45 µm membrane filter. A 100 µl aliquot of the clear extract was

diluted to 1 ml with the above mobile phase. Rat chow (2.45g) used in the experiment (#801700, Special diets services, Essex, UK) was powdered and extracted by sonication with 5 ml methanol. The extract was filtered and 100 µl aliquot was diluted to 1 ml with mobile phase.

Standard solution of ouabain exhibited a parent mass to charge ratio of m/z = 584.66, whereas digoxin standard solution shows a parent mass to charge ratio of m/z = 781.24. The ouabain pellet extract exhibited only a single parent mass to charge ratio m/z = 584.66 which corresponds to ouabain. The parent ion mass related to digoxin was absent in the pellet extract. The chow extract was also measured under the same conditions and no parent mass ions for ouabain or digoxin could be detected (S4-S5).

Statistical analysis

Most data were acquired with repeated measurements. However, ANOVA with repeated measures, which is commonly used in this situation, has a sphericity assumption. In our experience, the sphericity assumption is usually violated in telemetrically recorded variables. There are adjustments (*Greenhouse-Geisser, Huynh-Feldt*) to correct for violation of the sphericity assumption, but at the cost of a lower power. In addition, a correction factor computed on the full set of data does not apply well to tests based on only part of the data, so although the overall analysis might be protected, the multiple comparisons are not. Therefore, we have used a multivariate approach (MANOVA) to repeated measures designs, which does not have the sphericity assumption. Most experiments were designed to test main effects and interactions of 3 factors, i.e. between subjects main effect "group" with 2 levels (control, i.e. ouabain untreated animals and ouabain-treated animals), within subject (repeated measures) main effect "illumination" with 2 levels (dark, i.e. active period, when the animal room was not illuminated, and light period with increased frequency of sleeping episodes) and within subject (repeated measures) main effect "time/ouabain treatment" with 3 or 16 levels, which represent days when the telemetry data were recorded (16 levels) or the variability measures were calculated (3 levels). For the ouabain-treated rats, different levels of the main effect "time" simultaneously represent different doses of ouabain, thus the label "time/ouabain treatment". When the salt sensitivity was tested, the 4th main factor "salt intake" with 2 levels (low salt intake, high salt intake) was added.

The null-hypotheses were tested with multivariate tests for repeated measures (*Wilks, Pillai's, Hotelling, Roy's*). Because virtually in all cases the multivariable tests produced qualitatively identical results, only results from the *Wilks* multivariate test are included. If the null hypotheses were rejected, the significant main effects or interactions were explored with planned contrast and/or polynomial trend analysis, particularly when the main effect "time/ouabain treatment" had only three levels. If the main effect "time/ouabain treatment" had 16 levels, an omnibus post hoc *Tukey HSD* for unequal *n* (*Spjotvoll & Stoline*) test was used.

Data from experiments that did not involve repeated measurements (CGRP, cGMP, aldosterone in plasma) were analyzed with the one-way ANOVA. If the F-test was significant, *Tukey HSD* for unequal *n (Spjotvoll & Stoline)* was used to explore the significance of mean differences. Exceptionally, for the comparison of 2 groups, unpaired t-test for unequal sample size was used.

Distribution of samples was tested with the *Shapiro-Wilk W* test. If the *W statistic* was significant, the hypothesis that the respective distribution is normal was rejected and data were

logarithmically transformed before further processing. Homogeneity of variance was tested with the *Brown-Forsyth's* test.

Data are presented as means with corresponding standard deviation and/or 95% confidence intervals. Values of $P<0.05$ were considered significant. Statistica 12.0 (StatSoft, Inc., Tulsa, Oklahoma, USA) was used for all statistical testing with the exception of analysis of fold changes in gene expression. Exact information which statistical test was used to analyze particular data sets could be found below each table/figure.

Statistical testing of fold changes in gene expression could not be performed with "classical" tests because fold change data for the control group do not exist (fold change is defined as the ratio between gene expression in the experimental and the control group). Therefore, the pair-wise fixed reallocation randomization test was used. In this test, available data were randomly reshuffled (10 000 times) between the groups and the fold changes were calculated. To estimate the precision of fold changes assessment, 95% confidence intervals were calculated via bootstrapping method. Calculations were performed with the REST software [14].

References

1. Faul F, Erdfelder E, Buchner A, Lang AG (2009) Statistical power analyses using G*Power 3.1: tests for correlation and regression analyses. Behav Res Methods 41: 1149-1160.
2. Kuusela TA, Jartti TT, Tahvanainen KU, Kaila TJ (2002) Nonlinear methods of biosignal analysis in assessing terbutaline-induced heart rate and blood pressure changes. Am J Physiol Heart Circ Physiol 282: H773-H783.
3. Peng CK, Havlin S, Stanley HE, Goldberger AL (1995) Quantification of scaling exponents and crossover phenomena in nonstationary heartbeat time series. Chaos 5: 82-87.
4. Kamen PW, Tonkin AM (1995) Application of the Poincare plot to heart rate variability: a new measure of functional status in heart failure. Aust N Z J Med 25: 18-26.
5. Bertinieri G, Di RM, Cavallazzi A, Ferrari AU, Pedotti A, et al. (1988) Evaluation of baroreceptor reflex by blood pressure monitoring in unanesthetized cats. Am J Physiol 254: H377-H383.
6. Kim SY, Euler DE (1997) Baroreflex sensitivity assessed by complex demodulation of cardiovascular variability. Hypertension 29: 1119-1125.
7. Pagani M, Somers V, Furlan R, Dell'Orto S, Conway J, et al. (1988) Changes in autonomic regulation induced by physical training in mild hypertension. Hypertension 12: 600-610.
8. Matavelli LC, Zhou X, Varagic J, Susic D, Frohlich ED (2007) Salt loading produces severe renal hemodynamic dysfunction independent of arterial pressure in spontaneously hypertensive rats. Am J Physiol Heart Circ Physiol 292(2): H814-9.
9. Davis L, Kuehl M, Battey J (1994) Basic methods in molecular biology. Connecticut: Appleton & Lange. 777 p.
10. Al-Bader MD (2006) Estrogen receptors alpha and beta in rat placenta: detection by RT-PCR, real time PCR and Western blotting. Reprod Biol Endocrinol 4: 13.
11. Pfaffl MW (2001) A new mathematical model for relative quantification in real-time RT-PCR. Nucleic Acids Res 29: e45.
12. Ruijter JM, Ramakers C, Hoogaars WM, Karlen Y, Bakker O, et al. (2009) Amplification efficiency: linking baseline and bias in the analysis of quantitative PCR data. Nucleic Acids Res 37: e45.

13. Tuomi JM, Voorbraak F, Jones DL, Ruijter JM (2010) Bias in the Cq value observed with hydrolysis probe based quantitative PCR can be corrected with the estimated PCR efficiency value. Methods 50: 313-322.
14. Pfaffl MW, Horgan GW, Dempfle L (2002) Relative expression software tool (REST) for group-wise comparison and statistical analysis of relative expression results in real-time PCR. Nucleic Acids Res 30: e36.
